# A direct observation of ultrafast energy transfer, charge trapping and thermal quenching in inorganic phosphors

**DOI:** 10.1038/s41377-026-02433-z

**Published:** 2026-10-27

**Authors:** David Van der Heggen, Korneel Molkens, Jonas J. Joos, Daniel Rytz, Lucas C. V. Rodrigues, Pieter Geiregat, Philippe F. Smet

**Affiliations:** 1 LumiLab, Department of Solid State Sciences, Ghent University, Gent, 9000 Belgium; 2 PCN, Department of Chemistry, Ghent University, Gent, 9000 Belgium; 3 NOLIMITS, Core Facility for Non-Linear Microscopy and Spectroscopy, Ghent University, Gent, 9000 Belgium; 4 Faculty of Sciences, Antwerp Maritime Academy, Antwerpen, 2030 Belgium; 5 BREVALOR Sàrl, Les Sciernes, CH-1669 Switzerland; 6 Department of Fundamental Chemistry, Institute of Chemistry, University of São Paulo, São Paulo-SP, 05508-000 Brazil

**Keywords:** Fluorescence spectroscopy, Spectrophotometry

## Abstract

The functional properties of impurity-activated inorganic luminescent materials, or phosphors, used in lighting, sensors and radiation detection critically depend on their excited-state dynamics. These materials, and particularly persistent phosphors whose afterglow can last for hours after excitation ceases, are often regarded as systems dominated by slow emission processes. Yet, their excited-state dynamics are assumed to be governed by ultrafast events such as energy and charge transfer between dopants or nonradiative relaxation. Here, a combination of transient absorption and time-resolved luminescence spectroscopy is applied for the first time to capture the complete dynamics of these processes in benchmark SrAl_2_O_4_:Eu,(Dy), across an unprecedented time window from micro- down to picoseconds. It is found that nonradiative energy transfer between two distinct Eu^2+^ centers occurs within several nanoseconds after excitation, accelerates with increasing temperature and reaches an intrinsic minimum transfer time of 5 ps at high temperatures. Charge transfer from the Eu^2+^ activator to metastable trapping defects is roughly ten times faster than radiative decay, while, in contrast, reverse transfer following optical stimulation is much faster, taking place within 2–3 ps. Our study reveals that widely accessible pump–probe methods can resolve the ultrafast dynamics that govern seemingly slow phosphors, underscoring the need to bridge fast and slow timescales.

**Figure:**
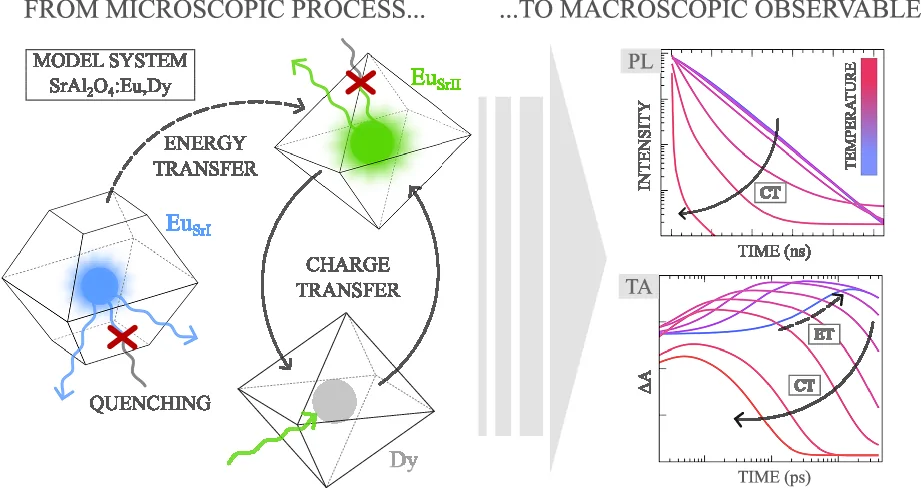

## Introduction

Impurity-activated luminescent materials, commonly referred to as phosphors, underpin a broad variety of technologies^1^ including solid-state lighting^2,3^, displays, lasing^4^, sensing^5–8^, and radiation dosimetry^9^. These materials consist of a wide-bandgap inorganic host that is intentionally doped with optically active ions. The dopants act as luminescent centers that absorb energy and subsequently emit photons in the ultraviolet^10^, visible, or infrared^11^ spectral range.

**Fig. 1:**
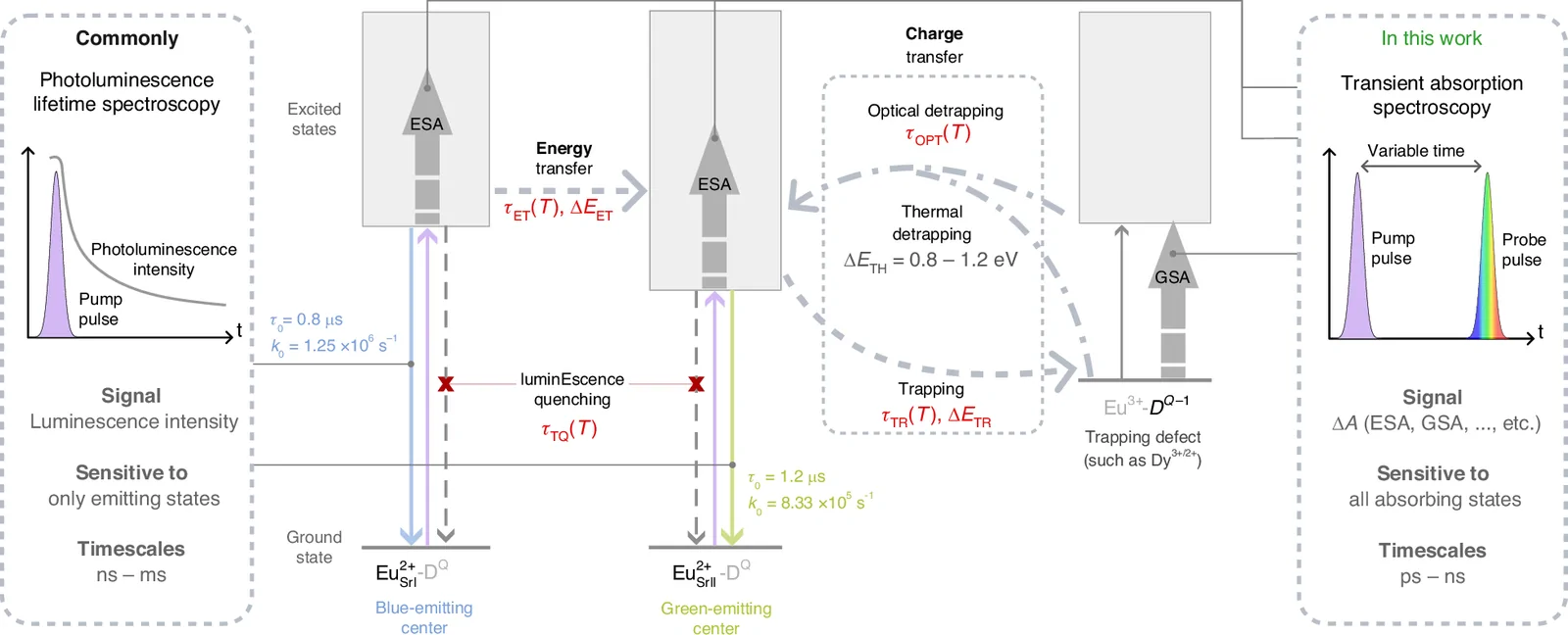
Schematic of photophysical processes in SrAl_2_O_4_:Eu,Dy. Excitation of Eu^2+^ is shown as violet upward-pointing arrows. Radiative processes probed by (time-resolved) luminescence spectroscopy are shown with solid (downward-pointing) arrows, while non-radiative processes, including luminescence quenching, energy transfer, and charge transfer, are indicated with dashed arrows. Contributions to absorption which are probed with transient absorption spectroscopy, such as excited-state absorption of ${{\rm{Eu}}}^{2+}$ (ESA) and ground-state absorption (GSA) of the trapping defect, are highlighted with bold grey arrows. Parameters such as characteristic times *τ* and activation energy barriers (Δ*E*) that are currently unknown and are addressed in this work are marked in red. This illustration is purely conceptual and does not imply any specific mechanism

Depending on the application, different metrics such as the thermal quenching profile^2^, quantum efficiency^12^, scintillation light yield^13^ or storage capacity^14^ are used to evaluate a material’s applicability. These macroscopic observables arise from a complex sequence of events occurring at the dopant level. However, direct access to these underlying mechanisms is limited, as phosphors are typically characterized using luminescence-based techniques that probe only the final outcome of this cascade (Fig. 1). As a result, insight into the processes that ultimately govern phosphor performance is often only indirectly obtained through simulations, which inherently introduce uncertainties. In this work, we present an experimental approach that enables direct observation of these ultrafast microscopic processes, and we apply this approach to the benchmark SrAl_2_O_4_:Eu, Dy phosphor in which, as outlined below, numerous processes such as quenching, energy and charge transfer take place simultaneously^14^.

**Fig. 2:**
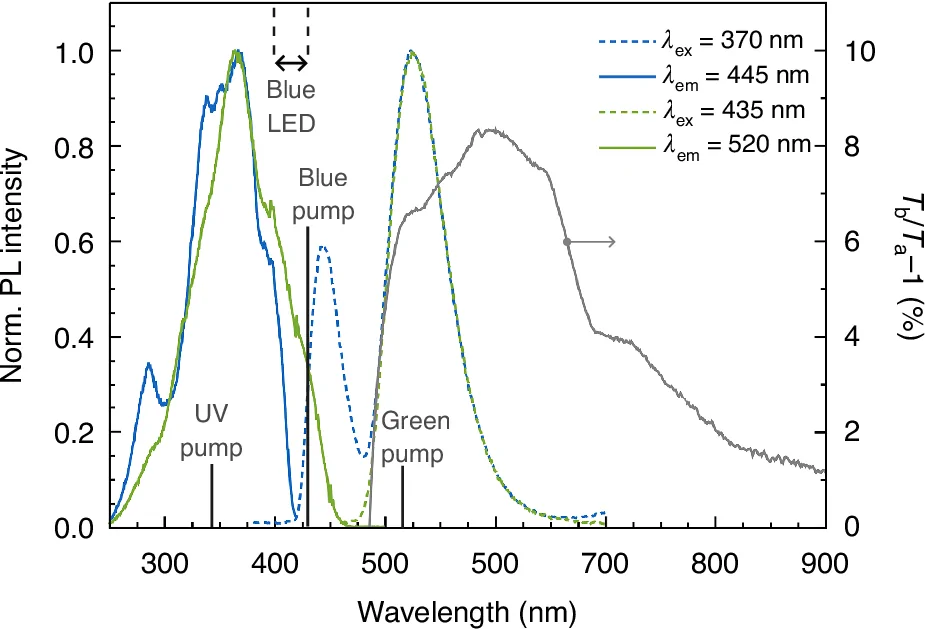
Eu^2+^ photoluminescence spectra and absorption spectrum of trapped charges in SrAl_2_O_4_:Eu,Dy. Photoluminescence (PL) emission (dashed traces) and excitation (solid traces) spectra of ${{\rm{Eu}}}_{{\rm{SrI}}}^{2+}$ (blue traces) and ${{\rm{Eu}}}_{{\rm{SrII}}}^{2+}$ (green traces) luminescence centers in SrAl_2_O_4_:Eu, Dy powder, recorded at 10 K. The ratio of the transmittance of the single crystal recorded at 75 K before (minimal trap filling, ${{\rm{T}}}_{b}$) and after (maximal trap filling, ${{\rm{T}}}_{a}$) exposure to blue light at 293 K (grey trace) represents the absorption of the trapped charges in ${{\rm{SrAl}}}_{2}{{\rm{O}}}_{4}$:Eu, Dy. The wavelengths of the different illumination sources (UV, blue and green pump, blue LED) used during the transient absorption spectroscopy are indicated with vertical lines

In SrAl_2_O_4_ the Eu^2+^ ions are incorporated in the host lattice on one of two inequivalent Sr-sites, thereby forming two distinct luminescent centers^15^. These Eu^2+^ luminescent centers absorb ultraviolet (UV) radiation and subsequently emit either blue (Eu^2+^ substituting on a SrI site, ${{\rm{Eu}}}_{{\rm{SrI}}}^{2+}$) or green (${{\rm{Eu}}}_{{\rm{SrII}}}^{2+}$) light (Figs. 1, 2)^16–18^. This photoluminescence only takes place while the phosphor is excited and at low temperature it decays with a lifetime (${\tau }_{0}$) of 0.8 µs or 1.2 µs, respectively^15^. Commercial phosphors used in solid-state lighting or displays operate on analogous principles, though they typically employ different host–dopant combinations optimized for stability and spectral conversion^19^.

However, many luminescent materials rely on interactions between multiple (luminescent) impurity centers or defects. One such interaction is energy transfer between different luminescent centers in which excitation energy is non-radiatively transferred from one luminescent center to another^20^. Energy transfer enables processes such as spectral sensitization, cooperative emission, and photon upconversion^21–23^. Energy transfer also occurs in SrAl_2_O_4_:Eu from the blue- to the green-emitting Eu^2+^ ions (Fig. 1)^24^.

In addition to energy transfer, many phosphors exhibit a second form of interaction between localized centers: a reversible charge transfer between dopants or between dopants and intrinsic defects^25–28^. In these materials, excitation of the luminescent center (the donor) does not necessarily lead to prompt photoluminescence but can instead be followed by charge transfer to a trapping defect (the acceptor), a process referred to as trapping^29^. While such a trapping defect, which can be both an intrinsic defect or an intentionally included impurity or dopant, often acts as a quenching center; in persistent or storage phosphors, it serves as a metastable electron or hole trap^30^. Upon stimulation of the phosphor with heat (thermal detrapping) or light (optical detrapping), a reverse charge transfer (starting from the acceptor side) can occur, enabling the released charge to recombine at the luminescent center and producing delayed emission (Fig. 1). For charge transfer processes to occur, an energy barrier must be overcome. In the case of thermal detrapping, the magnitude of this energy barrier is often referred to as the trap depth. While the concept of trap depth can vary with context, in this study, it is used to describe the effective activation energy associated with the thermal release of previously trapped charges.

In SrAl_2_O_4_:Eu, a limited amount of charge trapping occurs at intrinsic defects. However, the charge trapping can be spectacularly enhanced by several orders of magnitude through the introduction of a second dopant, namely Dy^31,32^. Upon codoping with Dy, the phosphor exhibits intense persistent luminescence that can last for hours after the excitation has stopped. Experimental evidence from X-ray absorption, diffuse reflectance, and photoluminescence spectroscopy confirms that while intrinsic defects may still play a role, the charge trapping in SrAl_2_O_4_:Eu,Dy is predominantly driven by introduction of Dy^3+^ and the delayed emission mainly arises from a reversible charge transfer between the dopant ions:^5,33–36^,

1 $${{\rm{Eu}}}^{2+}+{{\rm{Dy}}}^{3+}{{{\mathop{\longrightarrow }\limits^{{UV}/{blue}}}}\atop{{\mathop{\longleftarrow }\limits_{{k}_{B}T/{green}}}}}{{\rm{Eu}}}^{3+}+{{\rm{Dy}}}^{2+}$$

With the exception of the photoluminescence lifetimes and the energy barriers governing thermal detrapping of charges, a direct observation of the temperature-dependent dynamics, specifically the characteristic times and the associated activation energies (Fig. 1, red) of trapping, optical detrapping, energy transfer and quenching processes is still lacking. This introduces ambiguity in identifying which thermally or optically activated processes dominate under given conditions, since several pathways can produce similar macroscopic observables. As a result, the rational design and optimization of materials - particularly with respect to engineered trap depths, tailored energy-transfer routes, or mitigation of quenching channels - remain largely empirical rather than quantitatively guided.

One approach to probe the dynamics of these interactions uses photoluminescence lifetime spectroscopy, in which the phosphor is excited using a short pump pulse and the luminescence intensity is monitored over time (Fig. 1, left). However, this method is only sensitive to processes that ultimately yield radiative emission. While luminescence-based measurements offer the advantage of high sensitivity and a favorable signal-to-noise ratio, the radiative decay of the emitting species can be significantly slower than other excited-state processes. As a result, the signal strength may become too low to detect those processes effectively on the relevant experimental timescales.

Alternatively, transient absorption spectroscopy can be used to probe changes in the absorption of the phosphor^37^. Here, the luminescent material is excited with a pump pulse of a defined wavelength, after which changes in absorbance are monitored using a second, much weaker white-light probe pulse (Fig. 1, right)^38^. By varying the delay between the pump and probe pulses, changes in absorption can be tracked as a function of time with ultrashort (typically sub-ps) temporal resolution. During these measurements, both decreases and increases in absorption (*A*) relative to the unexcited state can be observed. A decrease in absorption, referred to as bleaching, appears as a negative Δ*A*, whereas processes that increase absorption, typically labeled photo-induced absorption, manifest as a positive Δ*A*. Transient absorption spectroscopy is especially powerful because it allows for the direct observation of all excited-state processes without relying on photon emission. As a result, it provides a much-needed complementary perspective to conventional time-resolved luminescence spectroscopy, allowing complex dynamic processes such as energy and charge transfer or luminescence quenching to be disentangled on ultrafast timescales.

In this work, we combine photoluminescence lifetime spectroscopy, which covers the nanosecond–millisecond regime, with transient absorption spectroscopy, which probes changes in absorption on the picosecond–nanosecond timescale, to determine the characteristic timescales of fast energy and charge transfer processes in slow inorganic luminescent materials for the first time. We thereby focus on SrAl_2_O_4_:Eu, Dy, a benchmark phosphor in which all radiative and non-radiative processes coexist, enabling a comprehensive measurement of energy and charge transfer dynamics within a single system.

## Results

### Steady state spectroscopy

The photoluminescence emission spectrum at 10 K (Fig. 2) consists of two emission bands peaking at 445 nm and 520 nm. These originate from the 4f^6^5d^1^→4f^7^ transition of Eu^2+^ substituting on a SrI and a SrII site, respectively^14^. The excitation spectra of these two emission bands are located in the UV-blue region of the spectrum and are very broad and featureless. They originate from transitions from the 4f^7^ ground state of Eu^2+^ to the dense 4f^6^5d^1^ excited-state manifold. Wavelengths below 420 nm excite both Eu^2+^ centers while excitation between 420 and 450 nm exclusively excites the ${{\rm{Eu}}}_{{\rm{SrII}}}^{2+}$ centers, resulting in isolated green emission. At higher temperatures, the spectra slightly broaden, and the blue emission intensity is suppressed due to a combination of energy transfer to the green-emitting site and thermal quenching.

### Transient absorption spectroscopy

**Fig. 3:**
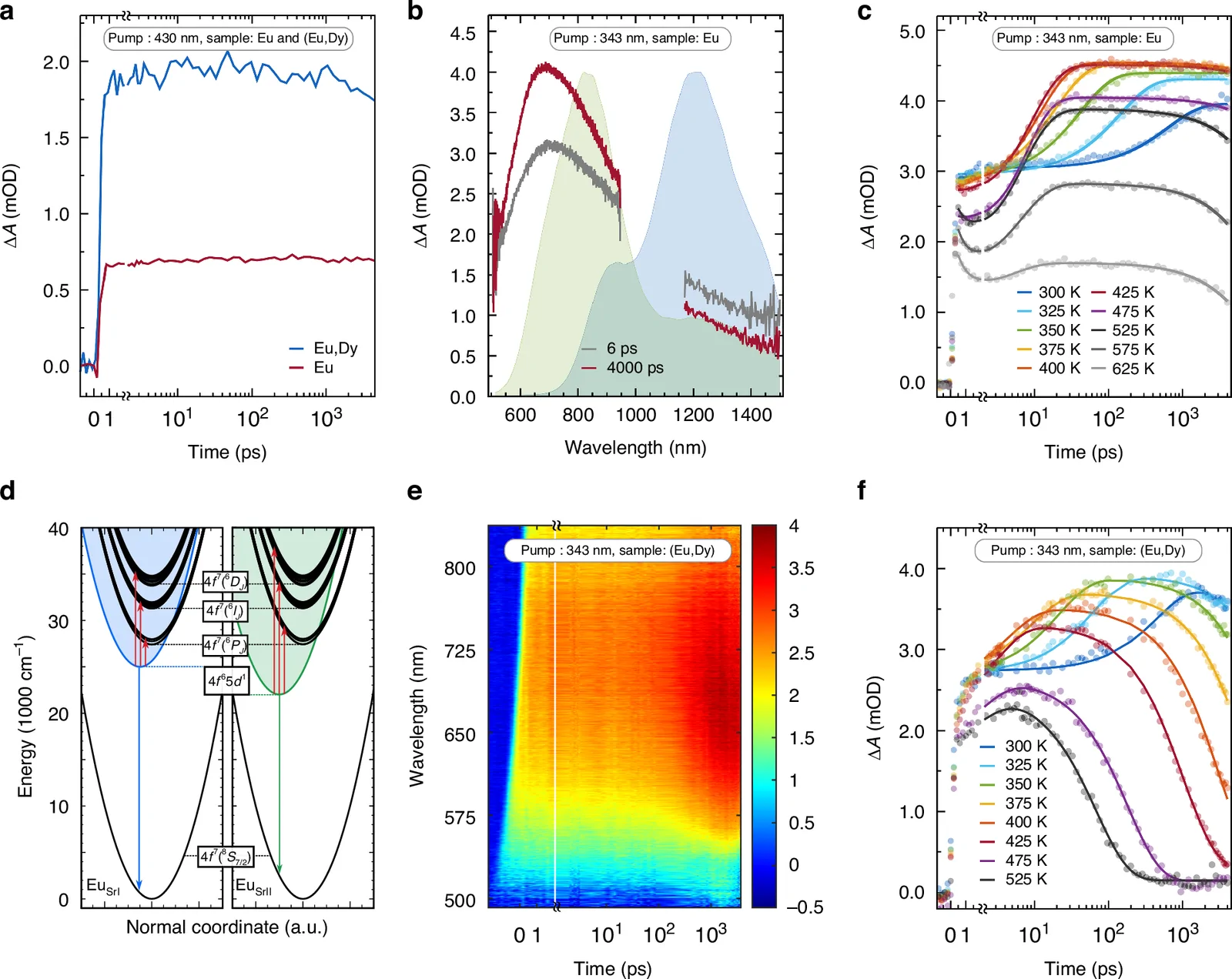
Transient absorption of the SrAl_2_O_4_:Eu, (Dy) single crystals recorded using a blue or UV pump. **a** Time traces showing ΔA(700 nm, t) recorded at 300 K on ${{\rm{SrAl}}}_{2}{{\rm{O}}}_{4}$:Eu and ${{\rm{SrAl}}}_{2}{{\rm{O}}}_{4}$:Eu, Dy with a blue pump (430 nm, 170 µJ/cm^2^, 1 kHz). **b** Absorbance spectra recorded at 300 K on ${{\rm{SrAl}}}_{2}{{\rm{O}}}_{4}$:Eu immediately and 4 ns after the UV pump pulse. The estimated shapes (Supplementary Information) of the ${{\rm{Eu}}}^{2+}$ excited-state absorption spectra are shown in blue (${{\rm{Eu}}}_{\mathrm{SrI}}^{2+}$) and green (${{\rm{Eu}}}_{\mathrm{SrII}}^{2+}$). **c** Time traces showing ΔA(700 nm, t) recorded at different temperatures on ${{\rm{SrAl}}}_{2}{{\rm{O}}}_{4}$:Eu with a UV pump (343 nm, 121 µJ/cm^2^, 0.5 kHz). The lines are a result of a fit using an exponentially increasing and two exponentially decreasing components (Table S2). **d** Schematic potential energy curves for Eu^2+^ incorporated on both Sr-sites in SrAl_2_O_4_. The black lines originate from the 4f^7^ configuration, whereas the blue/green color fills represent the dense 4f^6^5d^1^ manifolds for ${{\rm{Eu}}}_{\mathrm{SrI}}^{2+}/{{\rm{Eu}}}_{\mathrm{SrII}}^{2+}$. The red arrows indicate the 4f^6^ 5d^1^ → 4f^7^(^6^L_*J*_) (L = P, I, D) excited-state absorptions. The blue and green arrows indicate the 4f^6^ 5d^1^ → 4f^7^(^8^S_7/2_) emission. **e** Color map showing the resulting ΔA(*λ*,t) recorded at 300 K on SrAl_2_O_4_:Eu, Dy with a UV pump (343 nm, 186 µJ/cm^2^, 0.5 kHz). **f** Time traces showing ΔA(700 nm, t) recorded at different temperatures on SrAl_2_O_4_:Eu, Dy with a UV pump (343 nm, 186 µJ/cm^2^, 0.5 kHz). The lines are a result of a fit using an exponentially increasing and an exponentially decreasing component (Table S1)

First, transient absorption spectroscopy measurements were performed at room temperature on a SrAl_2_O_4_:Eu single crystal, without Dy^3+^ codoping, using a blue (430 nm) pump pulse (Fig. 3a), which selectively excites the green-emitting ${{\rm{Eu}}}_{{\rm{SrII}}}^{2+}$ centers (Fig. 2). Changes in absorbance were monitored using a visible (400–825 nm) and near-infrared (NIR, 1150–1500 nm) probe pulse, probing the absorption from 4 ps before up to 4 ns after the pump pulse. The blue pump pulse induces an immediate increase in absorption on timescales too fast to be resolved in the present setup (i.e. <270 fs; Fig. 3a). The resulting transient absorption spectrum is relatively broad, extending across the red and near-infrared regions and peaking at 665 nm. This absorption can be attributed to excited-state absorption of ${{\rm{Eu}}}_{{\rm{SrII}}}^{2+}$ ions in SrAl_2_O_4_ (Fig. 3b, d, Supplementary Information). The induced absorption remains essentially constant over the entire measured time window up to 4 ns after excitation (Fig. 3a). This behavior is consistent with the long excited-state lifetime of ${{\rm{Eu}}}_{{\rm{SrII}}}^{2+}$ in SrAl_2_O_4_, which is on the order of ~1.2 µs, meaning that within the first 4 ns the number of Eu^2+^ ions in the excited state is essentially constant. Furthermore, because the blue pulse selectively excites only the ${{\rm{Eu}}}_{{\rm{SrII}}}^{2+}$ site, no energy transfer to other centers is possible. Since this single crystal is activated solely with Eu and does not contain Dy codopants, charge transfer processes involving Eu ions and trapping defects are also expected to be minimal^31^.

To investigate the energy transfer dynamics, temperature-dependent transient absorption spectroscopy was performed on the same SrAl_2_O_4_:Eu single crystal but now using a UV (343 nm) pump, a wavelength that excites both Eu^2+^-centers (Fig. 2). At room temperature, the UV pulse induces an immediate absorbance increase of about 3 mOD (Fig. 3c). The transient absorption spectrum recorded directly after excitation is relatively featureless but shows a maximum near 735 nm (Fig. 3b). The absorbance in the visible region then continues to rise more slowly, reaching a maximum of 4 mOD at around 1.3 ns after the UV pulse. At the same time, the NIR absorbance decreases and the absorption maximum shifts to 665 nm (Fig. 3b). At longer times, this spectrum matches the one recorded for both single crystals using a blue pump pulse, solely exciting the ${{\rm{Eu}}}_{{\rm{SrII}}}^{2+}$ centers. As the temperature increases, the slow rise in intensity and the accompanying spectral changes accelerate, reaching a minimum rise time of ~5 ps at 475 K, which remains unchanged for higher temperatures. Above 475 K, the absorbance also decreases in two phases: an initial rapid drop within the first 3 ps after the UV pulse, followed by a much slower decrease around 1 ns after excitation. These decreases in absorbance can be linked to thermal quenching of the ${{\rm{Eu}}}_{{\rm{SrI}}}^{2+}$ and ${{\rm{Eu}}}_{{\rm{SrII}}}^{2+}$ excited states, respectively. The time traces (Fig. 3c) were fit using a model consisting of an exponentially increasing and two exponentially decreasing components, which allows to extract the characteristic times related to these non-radiative pathways (Table S2). The former information is not accessible through common luminescence spectroscopy at these elevated temperatures because the blue emission is strongly suppressed as a result of energy transfer to the green-emitting site combined with non-radiative quenching.

To investigate the charge transfer dynamics, the same experiment was performed on a SrAl_2_O_4_:Eu,Dy single crystal (Fig. 3e, f), which exhibits efficient charge trapping and has an integrated afterglow intensity that is 2500 times higher than that of the SrAl_2_O_4_:Eu single crystal (Fig. S8). At room temperature, the time-dependent absorbance closely resembles that of the SrAl_2_O_4_:Eu single crystal. An initial rapid increase to about 2.5 mOD is followed by a slower rise, accompanied by spectral changes. A subtle difference, however, is that after reaching a maximum at 1.3 ns, the absorbance in SrAl_2_O_4_:Eu, Dy decreases slightly. As the temperature increases, the rise in absorbance accelerates, similar to the behavior in SrAl_2_O_4_:Eu.

In contrast, though, the absorbance in SrAl_2_O_4_:Eu,Dy decreases much more rapidly with temperature: above 425 K it almost completely vanishes within 4 ns, leaving only a weak residual signal of about 0.15 mOD. The time traces (Fig. 3f) were fit using a model consisting of an exponentially increasing and one exponentially decreasing component, which allows to extract the characteristic times (Supplementary Information, Table S1).

Both single crystals thus exhibit a gradual rise in absorbance accompanied by changes in the absorption spectrum upon pumping in the UV. This behavior can be attributed to interactions between Eu ions and, as will be shown later, arises from energy transfer between the blue-emitting ${{\rm{Eu}}}_{\mathrm{SrI}}^{2+}$ and the green-emitting ${{\rm{Eu}}}_{\mathrm{SrII}}^{2+}$ sites. In contrast, the fast decrease in absorbance observed only in the SrAl_2_O_4_:Eu, Dy single crystal can be ascribed to charge trapping on the Dy codopant, as will be clarified below.

### Luminescence lifetime spectroscopy

**Fig. 4:**
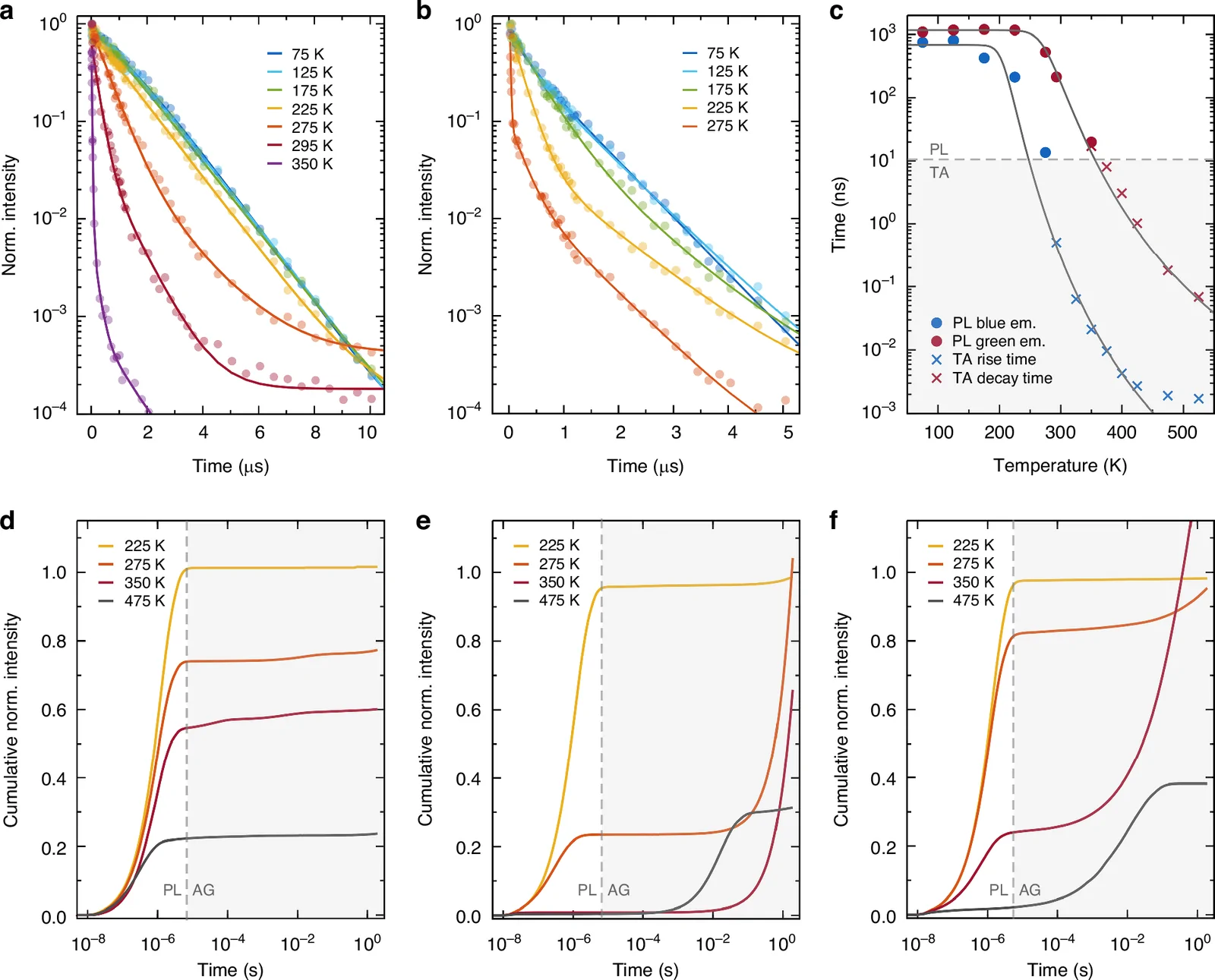
Luminescence lifetime measurements of Eu^2+^ in the SrAl_2_O_4_:Eu(,Dy) single crystals. Photoluminescence decay curves of the green (**a**) and blue (**b**) emission of the SrAl_2_O_4_:Eu, Dy single crystal at different temperatures after excitation with a low intensity (160 µJ/cm^2^, 10 Hz) 410 nm pulse. The solid lines represent the result of the fit to the experimental data (Supplementary information, Tables S3, S4) (**c**) The photoluminescence lifetimes (Fig. 4a, b and Tables S3, S4) and transient absorption rise and decay times (Fig. 3f, Table S1) as a function of temperature. The grey lines represent the fit with a single barrier model used to extract the relevant energy barriers. **d** Cumulative photoluminescence emission intensity of the SrAl_2_O_4_:Eu single crystal as a function of time after a low intensity (160 µJ/cm^2^, 0.5 Hz) 410 nm excitation pulse. Cumulative photoluminescence emission intensity of the SrAl_2_O_4_:Eu,Dy single crystal as a function of time after (**e**) low intensity (160 µJ/cm^2^, 0.5 Hz) and (**f**) high intensity (25.8 mJ/cm^2^, 0.5 Hz) 410 nm excitation pulses. The dashed vertical line divides the graph into two distinct regions: one corresponding to emission occurring shortly after the excitation pulse, attributable to photoluminescence (PL), and the other corresponding to emission at longer times after the excitation pulse, which originates from afterglow (AG)

The dynamics can also be studied through photoluminescence lifetime measurements (Fig. 4a, b). In these measurements the SrAl_2_O_4_:Eu, Dy single crystal is excited using a UV pulse (410 nm), after which the luminescence intensity is recorded as a function of time for each emission center. A low excitation intensity (160 μJ/cm^2^ at 10 Hz) was used to maintain low trap occupancy, ensuring that the charge transfer pathways remained active. This condition also facilitates a direct comparison with the results obtained from transient absorption measurements, which were recorded under similar excitation intensities. The experimental results were fit using different prescriptions to extract values of the characteristic lifetimes (Supplementary Information, Tables S3, S4). At 75 K, the blue and green emissions of the SrAl_2_O_4_:Eu, Dy single crystal have a lifetime of 750 ns and 1100 ns, respectively. The difference between the two lifetimes can mainly be ascribed to the wavelength dependency of the decay time and is in line with earlier reports on SrAl_2_O_4_:Eu, Dy powders^24,39^. As temperature is increased, the decay profiles of the blue and green emission bands become increasingly non-exponential and the photoluminescence lifetimes shorten to 13 ns (at 275 K) and 20 ns (at 350 K), respectively.

### Energy-transfer dynamics

**Fig. 5:**
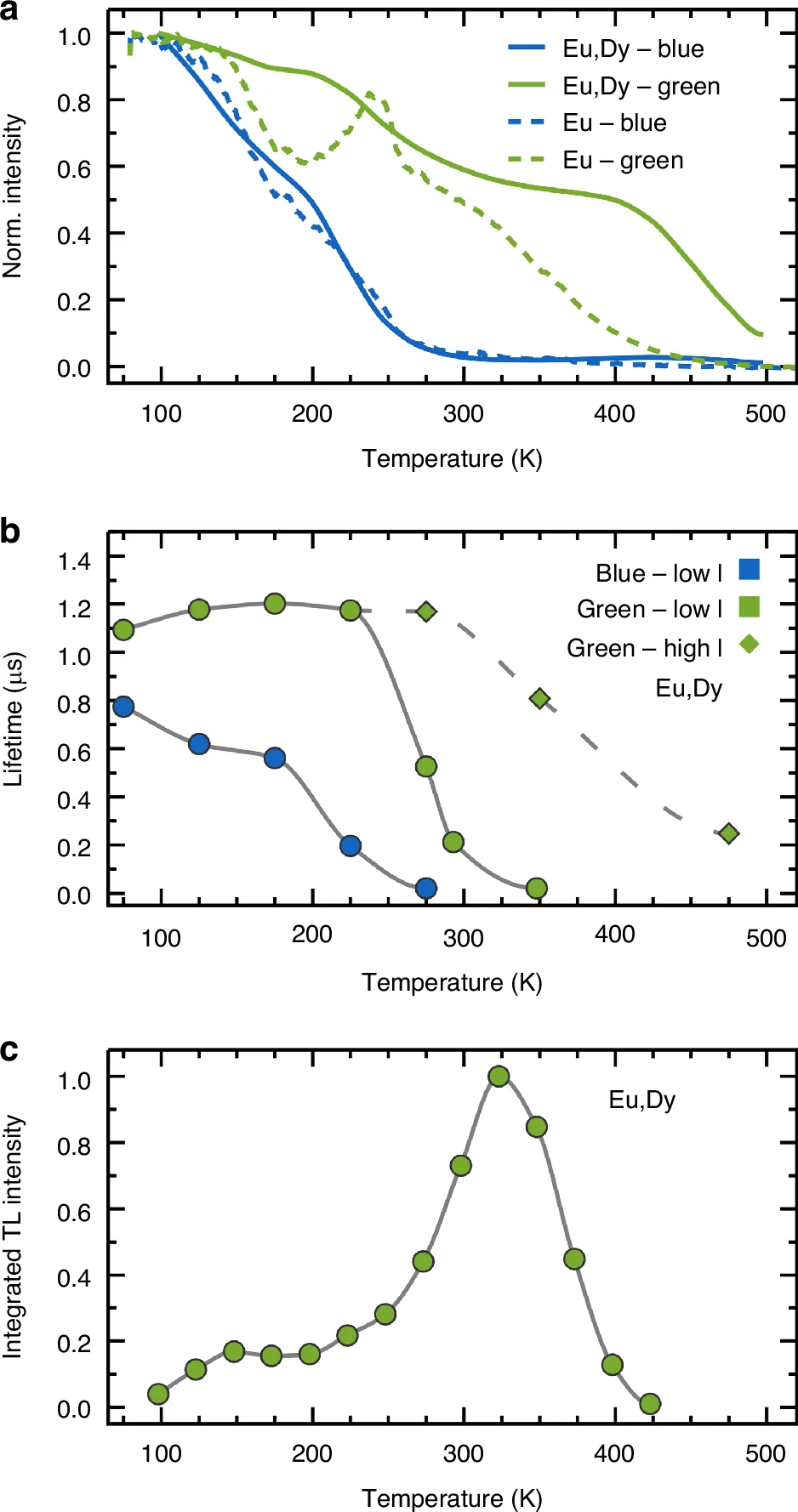
Effect of temperature on the luminescence intensity, luminescence lifetime and trap filling. **a** Thermal quenching profiles recorded under pulsed 410 nm excitation (10 Hz, 31.3 mJ/cm^2^). **b** Photoluminescence lifetime of the blue and green emission of the SrAl_2_O_4_:Eu, Dy single crystal recorded under pulsed (10 Hz) 410 nm excitation with different intensity (low intensity: 160 μJ/cm^2^; high intensity: 25.8 mJ/cm^2^). **c** Integrated thermoluminescence intensity after excitation with 410 nm light at different temperatures

A shortening of photoluminescence lifetime with increasing temperature is usually a consequence of non-radiative de-excitation pathways that open up or existing quenching processes that accelerate, thereby accelerating the depopulation of the ${{\rm{Eu}}}^{2+}$ excited state leading to an observed lifetime which is shorter than the radiative lifetime in the absence of other de-excitation routes. Since most of these de-excitation pathways lead to quenching of the luminescence, the decrease in photoluminescence lifetime with higher temperature is usually accompanied by a decrease in photoluminescence intensity. For the blue emission, the decrease in photoluminescence lifetime indeed aligns with the thermal quenching profile (Fig. 5a, b), exhibiting an identical temperature dependence. The driving mechanism behind this decrease in blue emission intensity is known: energy transfer from the blue-emitting ${{\rm{Eu}}}_{\mathrm{SrI}}^{2+}$ to the green-emitting ${{\rm{Eu}}}_{\mathrm{SrII}}^{2+}$. The existence of non-radiative energy transfer between these two nonequivalent ${{\rm{Eu}}}^{2+}$-centers has been demonstrated through concentration- and temperature-dependent lifetime measurements^24^ in powder phosphors. As a consequence of this energy transfer, the low-temperature photoluminescence decay profiles of the green emission (Fig. 4a) exhibit a slight increase in intensity with a rise time that is equal to the photoluminescence lifetime of the blue emission (Fig. 4b). In the thick translucent crystals studied here, it cannot be excluded that a part of the energy transfer is also radiative.

From a comparison of the dynamics observed in transient absorption and luminescence lifetime spectroscopy at different temperatures (Fig. 4c), it is clear the shortening of the blue photoluminescence lifetime and the slow rise in absorbance observed in transient absorption spectroscopy after excitation of the SrAl_2_O_4_:Eu and SrAl_2_O_4_:Eu, Dy single crystals with a UV pulse, are interconnected (Fig. 4c). Hence it can be concluded that the latter also originates from energy transfer between the two nonequivalent ${{\rm{Eu}}}^{2+}$-sites. The experimentally determined ${{\rm{Eu}}}_{\mathrm{SrI}}^{2+}$ excited-state lifetime (Fig. 4c) decreases by no less than six orders of magnitude in the measured temperature range. At room temperature, the transfer occurs on a timescale of about 1 ns and accelerates with increasing temperature. A minimum transfer time of 5 ps is reached at 475 K, beyond which further temperature increases do not reduce the transfer time. At the same time, the spectral overlap S(T) between the (electron-vibrational) emission spectrum of the donor ${F}_{D}(E)$ and the excitation spectrum of the acceptor ${F}_{A}(E)$,

2 $$S\left(T\right)=\int {F}_{D}\left(E,T\right){F}_{A}\left(E,T\right){E}^{-4}{\mathrm{d}}{E}$$

increases only weakly and approximately linearly with temperature (Fig. S7)^40^. Consequently, the temperature dependence of the energy transfer kinetics cannot be explained by spectral overlap alone (Förster model for energy transfer)^20,41^, implying that at least one of the steps during the energy transfer is thermally activated. The temperature dependence of the observed lifetimes τ(T) can be described using a phenomenological single barrier model^42^

3 $$\tau (T)=\frac{{\tau }_{0}}{1+A\exp \frac{-\Delta E}{{k}_{B}T}}$$

which yields an activation energy for energy transfer of $\Delta {E}_{{ET}}=0.45\,{\rm{\ eV}}$ ($A=6.73\times {10}^{10}$, ${\tau }_{0}=532\,{\rm{\ ns}}$).

The energy transfer can also explain the spectral changes observed in the transient absorption spectroscopy measurements (Fig. 3b). As energy transfer causes a simultaneous decrease in the ${{\rm{Eu}}}_{\mathrm{SrI}}^{2+}$ excited-state population and an increase in the ${{\rm{Eu}}}_{\mathrm{SrII}}^{2+}$ population, the transient absorption spectrum, determined by the excited-state absorption of the two ${{\rm{Eu}}}^{2+}$-centers, also evolves over time. Based on the known energies of the involved states, the excited-state absorption spectra of both ${{\rm{Eu}}}^{2+}$ centers in SrAl_2_O_4_ can be estimated (Supplementary Information)^18,39,43^. These spectra are governed by electric-dipole transitions from the lowest 4f^6^5d^1^ excited state to higher-lying multiplets of the 4f^7^ configuration (^6^P_*J*_, ^6^I_*J*_, and ^6^D_*J*_, Fig. 3d). Because the lowest 4f^6^5d^1^ excited state of ${{\rm{Eu}}}^{2+}$ is higher in energy for the ${{\rm{Eu}}}_{\mathrm{SrI}}^{2+}$ site than for the ${{\rm{Eu}}}_{\mathrm{SrII}}^{2+}$ site and because the 4f^7^ multipets are negligibly affected by the different chemical environment, the excited-state absorption spectrum of ${{\rm{Eu}}}_{\mathrm{SrI}}^{2+}$ is red-shifted by about 3500 cm^−1^ with respect to that of ${{\rm{Eu}}}_{\mathrm{SrII}}^{2+}$. Comparison of the estimated excited-state absorption spectra with the transient absorption spectra (Fig. 3c) confirms that the observed spectral changes indeed originate from energy transfer between the two ${{\rm{Eu}}}^{2+}$-sites.

### Charge transfer dynamics

For the green emission band in SrAl_2_O_4_:Eu, Dy (centered around 520 nm), the situation is entirely different, and the photoluminescence lifetime already decreases considerably around 225 K while the thermal quenching only sets in above 400 K (Fig. 5a, b). This implies the existence of a depopulation pathway of the ${{\rm{Eu}}}^{2+}$ excited state that does not lead to strong losses in emission intensity, as was the case for the blue emission center. Instead, the decrease in lifetime appears to align with the temperatures at which trapping becomes thermally activated, suggesting that a charge transfer to a trapping defect is responsible for the shortening of the luminescence lifetime. To illustrate this, the single crystal was exposed to 410 nm light at different temperatures, after which it was heated at a linear rate to stimulate the electron back transfer, and the resulting thermally stimulated luminescence was collected (Figs. 5c, S2)^44^. Below room temperature, there is only limited trapping, but as the temperature is further increased to 275 K, the integrated thermoluminescence intensity suddenly increases as well. This indicates that the charge trapping in SrAl_2_O_4_:Eu, Dy is thermally activated, i.e. an energy barrier needs to be overcome before the charge transfer can take place. Around 350 K, the integrated thermoluminescence intensity decreases again, which is a consequence of the accelerated detrapping rate at elevated temperatures, resulting in a lower overall trap occupation.

Here too, the change in luminescence lifetime of the green-emitting ${{\rm{Eu}}}_{\mathrm{SrII}}^{2+}$ center with increasing temperature aligns with the change in transient absorption decay time observed in the SrAl_2_O_4_:Eu, Dy single crystal (Fig. 4c), again indicating that both observations are interconnected. The depopulation of the ${{\rm{Eu}}}^{2+}$ excited state through charge transfer to a trapping defect is therefore directly responsible for the fast decrease in absorbance observed in SrAl_2_O_4_:Eu, Dy after excitation with the UV pump pulse (Fig. 3e, f). At room temperature, the charge transfer between ${{\rm{Eu}}}^{2+}$ and the trapping defect occurs on a timescale of ~100 ns. When the temperature is raised to 525 K, this process accelerates dramatically, and the absorption almost completely vanishes within the first 100 ps. Also here, the temperature dependence can be modeled using a single barrier model (Eq. 3) yielding an activation energy for charge trapping $\Delta {E}_{{TR}}=0.49{\rm{\ eV}}$ ($A=1.03\times {10}^{9}$, ${\tau }_{0}=1181{\rm{\ ns}}$).

The residual absorption observed at 525 K on longer timescales closely matches the ground-state absorption spectrum of Dy^2+^ in SrAl_2_O_4_:Eu, Dy powders (Fig. S3)^35^, confirming that charge transfer from Eu^2+^ to Dy^3+^ at elevated temperatures indeed occurs within the first 100 ps following the 343 nm pump pulse.

The fact that charge transfer from the Eu^2+^ excited state to the Dy occurs on a timescale (100 ns at 300 K) shorter than the typical radiative decay (~1.1 µs) of Eu^2+^ implies that charge trapping can effectively compete with radiative relaxation. In fact, based on these decay times, the branching ratio for the charge transfer process following Eu^2+^ excitation is about 92%, which means that the former process is roughly 12 times more likely than the latter. This confirms the findings of Romero et al.^45^, who found from a frequency-domain characterization of the luminescence in SrAl_2_O_4_:Eu, Dy that trapping is 17 times more likely than radiative de-excitation.

Furthermore, it can be concluded that all Eu^2+^ ions that are excited during the transient absorption measurement participate in the charge transfer and charge trapping process. If this were not the case, the fraction of Eu^2+^ ions not involved in trapping would result in a residual Eu^2+^ excited-state absorption persisting for several µs, corresponding to the typical photoluminescence lifetime of Eu^2+^ in SrAl_2_O_4_ without Dy.

This is confirmed by taking a closer look at the time-resolved, cumulative photoluminescence intensity which indicates how much light is emitted by the phosphor during the first two seconds after an excitation pulse with UV light (410 nm). For the SrAl_2_O_4_:Eu single crystal (Fig. 4d), which exhibits almost no trapping, most of the light is emitted within the first 5 µs after the UV pulse (or roughly five times the photoluminescence lifetime). It is clear that the luminescence lifetime shortens and the emission intensity decreases with increasing temperature, indicative of thermal quenching, but there is no significant emission intensity at longer times. It can hence be concluded that the emission intensity can almost be entirely attributed to the normal photoluminescence process and that excitation of Eu^2+^ is followed by immediate de-excitation to the Eu^2+^ ground state. For the SrAl_2_O_4_:Eu, Dy single crystal the situation is entirely different (Fig. 4e). At 225 K, where trapping is not yet thermally activated, the decay profile is similar to the one obtained on SrAl_2_O_4_:Eu. At temperatures where trapping becomes thermally activated, however, the decay profile changes spectacularly. The contribution of the luminescence observed during the first 5 µs after the excitation pulse decreases with increasing temperature and nearly all of the crystal’s luminescence is emitted at much larger times, at least several ms after excitation. This confirms that almost all excited Eu^2+^ ions transfer a charge to a trapping defect and the luminescence is only observed after detrapping and recombination of the transferred charge at the Eu^2+^ ion. This accounts for why, at low excitation intensities, the observed photoluminescence decay time decreases with temperature more rapidly than expected, while the single-crystal emission intensity does not exhibit the same temperature dependence. As temperature increases further, the overall intensity decreases due to thermal quenching and the emitted afterglow also shifts to shorter timescales due to an accelerated thermally-driven detrapping.

The observed lifetimes are also strongly dependent on the excitation intensity. When the excitation intensity is increased (Fig. 4f), the contribution of Eu^2+^ emission on short timescales becomes more prominent relative to the afterglow, suggesting an enhanced role of either conventional photoluminescence or optically stimulated luminescence induced by excitation light^46^ as trap filling approaches saturation. The latter process has been demonstrated to play a significant role during the excitation of SrAl_2_O_4_:Eu, Dy phosphors^46^ and should also be taken into account in this context, as the interval between successive excitation pulses is too brief to permit any substantial depletion of the trapped charges.

**Fig. 6:**
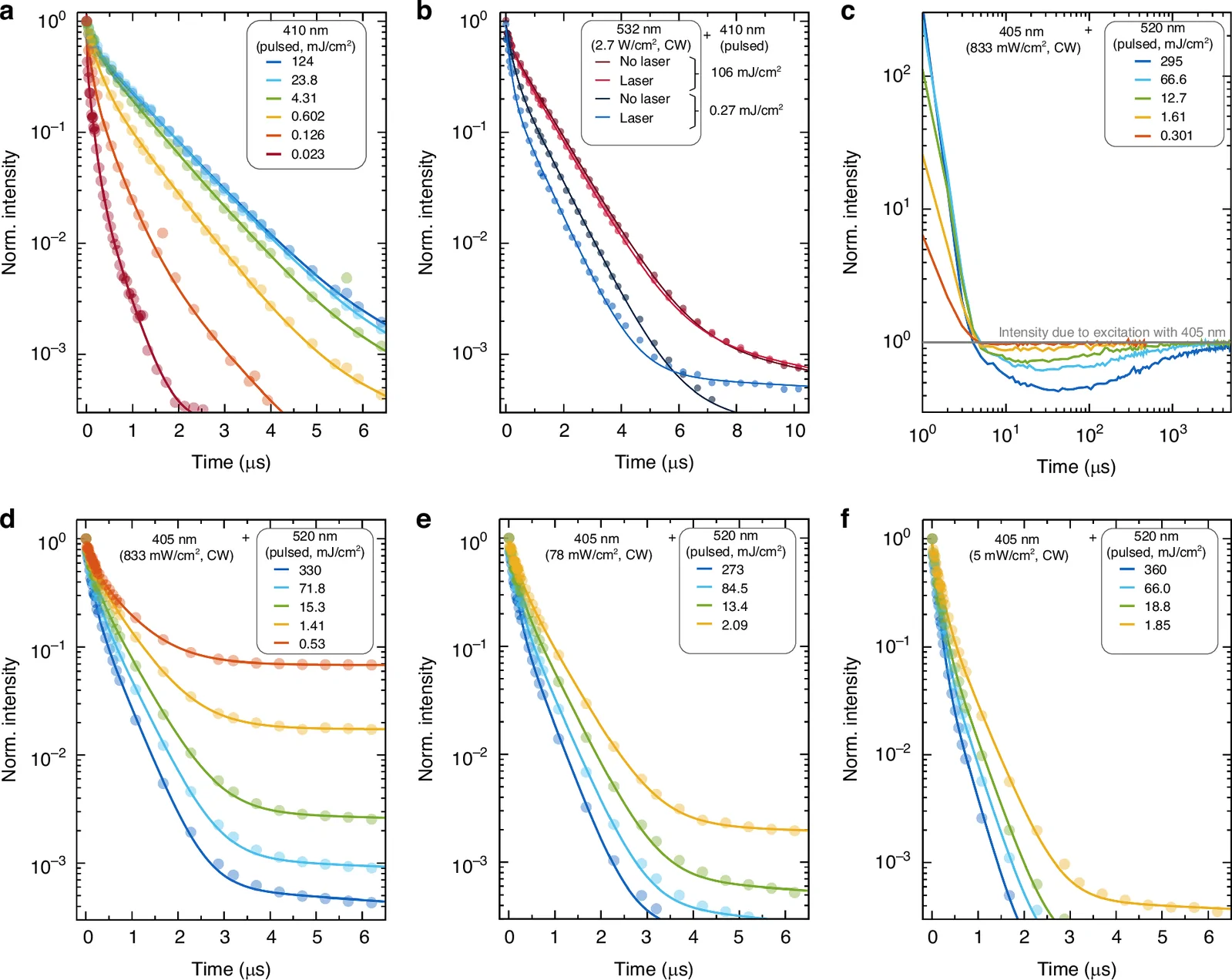
Luminescence lifetime measurements of ${{\rm{Eu}}}_{{\rm{SrII}}}^{2+}$ in the SrAl_2_O_4_:Eu, Dy single crystal under different combinations of excitation and optical stimulation. **a** The luminescence decay curves under pulsed excitation with 410 nm (10 Hz, different pulse intensities). **b** The luminescence decay curves under excitation with 410 nm (10 Hz, different pulse intensities) and continuous stimulation with a 532 nm laser. **c** The luminescence decay curves under continuous high power excitation with a 405 nm laser and pulsed stimulation with a 520 nm laser (10 Hz, different pulse intensities). **d**–**f** The luminescence decay curves under continuous high, medium and low power excitation with a 405 nm laser and pulsed stimulation with a 520 nm laser (10 Hz, different pulse intensities). All experiments were performed at 300 K

To distinguish between photoluminescence and optically stimulated luminescence, the luminescence lifetime was recorded under two distinct conditions: either using light that can induce trapping, in which case we refer to the process as excitation of the phosphor, or using light that can induce optically stimulated detrapping, in which case we refer to the process as stimulation. In a first experiment the luminescence lifetimes were recorded following direct excitation of Eu^2+^ with 410 nm light (similar to the results obtained in Fig. 4). In a second experiment the crystal was continuously illuminated with a 405 nm laser to maintain a steady trap population after which it was exposed to pulsed 520 nm light which is not able to excite the Eu^2+^ centers in the ground state but can induce optically stimulated detrapping^32^, which in turn populates the Eu^2+^ excited state. In both cases, the light output was recorded as a function of time following the 410 nm and 520 nm pulses, respectively. These measurements were carried out using different excitation and stimulation pulse intensities, and in both cases, it was found that the observed luminescence lifetimes were strongly dependent on the intensity of the excitation or stimulation pulses (Fig. 6 and Tables S5, S6).

As the intensity of the 410 nm excitation pulses increases, more traps are filled and the observed luminescence lifetime increases (Fig. 6a, Table S3). Ultimately, at a pulse intensity of 23.8 mJ/cm^2^, the photoluminescence lifetime approaches the intrinsic 1.1 µs lifetime of ${{\rm{Eu}}}_{\mathrm{SrII}}^{2+}$ recorded in singly doped SrAl_2_O_4_:Eu crystals. If, in addition, the single crystal is continuously illuminated with green 532 nm light during photoluminescence lifetime measurements under pulsed 410 nm excitation, optically stimulated detrapping occurs (Fig. 6b) in between the 410 nm pulses, lowering the trap occupancy and the photoluminescence lifetime again decreases. This effect is most pronounced at low excitation (410 nm) intensities, where trap emptying via thermal and optical stimulation can effectively compete with trap filling. With increasing excitation intensities, however, the traps remain mostly occupied, and the influence of optical stimulation becomes less significant.

These variations in recorded luminescence lifetime are hence directly linked to the trap occupancy within the single crystal. As trap occupancy increases, non-radiative depopulation of the Eu^2+^ excited state via charge transfer to a trap becomes less significant, causing the observed luminescence lifetime to approach the intrinsic lifetime of Eu^2+^, similar to the one observed in the SrAl_2_O_4_:Eu crystal. At these high excitation intensities the temperature dependency of the green ${{\rm{Eu}}}_{\mathrm{SrII}}^{2+}$ emission follows the recorded thermal quenching profile (Fig. 5b).

When measuring luminescence lifetimes following pulsed 520 nm optical stimulation under continuous 405 nm excitation (to ensure a constant average trap occupancy over time), a different trend emerges: the luminescence lifetime decreases with increasing intensity of the 520 nm stimulation pulse (Fig. 6d–f and Table S6) and increases with the intensity of the continuous 405 nm excitation.

The changes in luminescence lifetimes observed after optical stimulation can similarly be attributed to variations in trap occupancy. As the intensity of the stimulation pulse increases, more traps are emptied, resulting in lower trap occupancy. This, in turn, enhances the non-radiative depopulation of the Eu^2+^ excited state via charge transfer to an available (empty) trap, thereby shortening the luminescence lifetime. Supporting this interpretation, the luminescence intensity recorded up to 5 ms after the stimulation pulse (Fig. 6c) shows that, at the highest stimulation intensity, the emission falls below the baseline level observed under continuous 405 nm excitation alone. At high stimulation intensities, the observed intensity drops by at least 50% following the stimulation pulse, which suggests that a significant fraction of the traps is emptied. In contrast, at low stimulation intensities, the intensity drop amounts to only a few percent. However, the exact proportion cannot be determined because the contributions of photoluminescence, optically stimulated luminescence, and persistent luminescence cannot be separated. When the trap filling rate is increased by raising the excitation intensity (Fig. 6d–f), trap occupancy rises, reducing the effectiveness of the charge transfer depopulation pathway. As a result, the luminescence lifetime increases. Nevertheless, even at low stimulation intensities and high excitation intensities, the Eu^2+^ luminescence lifetime after optical stimulation remains approximately 0.7 µs, which is still significantly shorter than the 1.1 µs photoluminescence lifetime.

These results have significant implications for understanding the interaction between Eu^2+^ ions and trapping defects in the crystal. As described before, the photoluminescence lifetime measurements and the transient absorption experiments indicate that all Eu^2+^ ions are involved in the charge trapping process, rather than only a subset. Moreover, the observation that the photoluminescence lifetime increases with excitation intensity, as trap saturation is reached, and that the contribution of photoluminescence relative to afterglow rises again under high excitation conditions, suggests that multiple Eu^2+^ ions are coupled to the same trapping defect: when all accessible traps become occupied, further charge transfer is no longer possible, and the remaining Eu^2+^ ions relax radiatively via regular photoluminescence.

This process is stochastic, meaning that at any given time, a different subset of Eu^2+^ ions will have transferred a charge to the trapping defects, and consequently, a different subset will contribute to the observed photoluminescence under high excitation conditions. Because the intrinsic photoluminescence lifetime of 1.1 µs, matching the lifetime of Eu^2+^ in singly doped SrAl_2_O_4_:Eu, is fully recovered at high excitation intensities, it can be concluded that all Eu^2+^ ions exhibit equivalent luminescent properties and are subject to similar quenching environments. The observation that the optically stimulated luminescence lifetime at room temperature (0.7 µs) is significantly shorter than the photoluminescence lifetime (1.1 µs) is thus a consequence of the fact that Eu^2+^ ions that participate in optically stimulated luminescence are inherently coupled to an empty trapping defect and that the depopulation of the Eu^2+^ excited state through a charge transfer back to the trapping defect shortens the observed luminescence lifetime. This contrasts with previous reports^47,48^, which suggested that the reduced optically stimulated luminescence lifetime was due to enhanced quenching from defects in close proximity to the Eu^2+^ ion involved in trapping.

The optically induced detrapping by green light also allows for the use of transient absorption spectroscopy to study the dynamics of the detrapping process, i.e., the electron transfer from the trap, mostly the Dy codopant, back to Eu. For this, the single crystal was exposed to continuous illumination with a blue LED (420 nm) to maintain a certain trap occupation, after which the sample was subjected to a green (515 nm) pump pulse, which induces optically stimulated detrapping (Fig. 2).

The magnitude of the observed change in absorbance following the green pump pulse is strongly dependent on the intensity of the continuous blue illumination (Fig. S4). The absorbance increases with increasing blue light intensity, reaching a maximum of 0.5 mOD, beyond which a further increase in blue light intensity does not lead to additional changes in absorbance, likely due to a saturation in the trap filling. No change in absorbance is observed if the SrAl_2_O_4_:Eu, Dy crystal is not exposed to blue light, nor in the singly doped SrAl_2_O_4_:Eu single crystal. This allows for an unambiguous assignment of the observed absorbance changes to trap-related processes.

The transient absorption measurements on the SrAl_2_O_4_:Eu, Dy crystal under excitation with UV (343 nm) and green (515 nm) pump excitation produce absorbance spectra of a similar shape (Fig. S5b). Given that the absorbance spectrum observed after UV excitation (Fig. 3c) has been assigned to excited-state absorption of Eu^2+^, the data indicate that optical stimulation also quickly populates the Eu^2+^ excited state. A possible contribution of excited-state absorption of Dy^2+^ is expected to be very limited because, in contrast to Eu^2+^ where multiple spin-allowed 4f^6^5d^1^ → 4f^7^ transitions occur (see Figs. 3d and S1), no spin-allowed 4f^9^5d^1^ → 4f^10^ transitions exist in Dy^2+^ due to the spin-septet nature of the lowest 4f^9^ 5d^1^ states, giving rise to a significantly lower transition probability^49,50^. The temporal response of the transient absorption (Fig. S5a), however, shows a distinct difference: following the green pump pulse at room temperature, the absorbance rise is delayed by approximately 2 ps. This observation finally constrains the charge transfer from Dy^2+^ to Eu^3+^ to a timescale of only a few picoseconds after optical stimulation of the trapped electron.

Our findings show that standard pump–probe spectroscopy can directly access the ultrafast dynamics underlying apparently slow phosphors, making it possible to connect fast excitation processes to long-lived emission. This strategy has allowed us to determine, for the first time, the relevant kinetic rates and energy barriers governing the different processes, providing a quantitative foundation that was previously unavailable.

## Discussion

By combining transient absorption spectroscopy with time-resolved luminescence spectroscopy, we have mapped the characteristic timescales of energy and charge transfer processes in SrAl_2_O_4_:Eu,(Dy) phosphors. At room temperature, energy transfer between blue- and green-emitting Eu^2+^ sites occurs on the nanosecond scale, while charge transfer from Eu^2+^ to trapping defects is nearly an order of magnitude faster than Eu^2+^ radiative decay (~1.1 µs), taking place within ~10^2^ ns. This implies that, following Eu^2+^ excitation, a charge transfer between the Eu^2+^ activator and a trapping defect is about twelve times more likely than regular radiative decay. At elevated temperatures, this charge transfer accelerates to ~10^2^ ps, and the subsequent absorption spectrum reveals the presence of Dy^2+^, confirming that charge transfer to the codopant is completed on this ultrafast timescale. Under 515 nm optical stimulation, which drives the reverse charge transfer, the Eu^2+^ excited state is repopulated with a characteristic delay of 2–3 picoseconds.

Luminescence lifetime measurements further show that both photoluminescence and optically stimulated luminescence lifetimes strongly depend on trap occupancy, with lifetimes decreasing significantly as trap populations are depleted. Notably, the optically stimulated luminescence lifetime reaches a maximum of 0.7 µs, shorter than the ~1.1 µs photoluminescence lifetime. This difference is attributed to the coupling of the optically re-populated Eu^2+^ ion to an empty trap, which enables efficient charge transfer and results in faster decay. These findings highlight the importance of retrapping in persistent luminescence of SrAl_2_O_4_:Eu,Dy and suggest that simple first-order kinetic models may be insufficient for describing trapping and detrapping behavior, at least when optical stimulation is relevant.

The experimental results presented herein are compatible with both local (i.e. direct transfer between luminescent center and trapping defect) and global (i.e. involving conduction band levels) charge transfer mechanisms. However, the current data do not provide a basis for distinguishing definitively between these two models. Further investigation is necessary to resolve this question.

This work establishes ultrafast pump-probe spectroscopy as a versatile and powerful tool for elucidating the fundamental non-radiative quenching, charge, and energy transfer pathways that govern excited-state dynamics in inorganic phosphors. As such, it complements the available tool set of spectroscopic techniques, including time-resolved X-ray absorption and electron paramagnetic resonance techniques which allow to directly observe valence state changes. Moreover, it lays a methodological foundation for applying this approach to other luminescent materials, including organic compounds, hybrid organic-inorganic systems, quantum dots, and perovskites, each of which may involve distinct underlying physical mechanisms.

By capturing these processes on their intrinsic picosecond timescales, the approach presented here provides a way to directly connect microscopic processes and macroscopic observables such as luminescence efficiency, thermal quenching behavior and decay kinetics. Hence, it opens the path toward a deeper understanding and the rational design and optimization of inorganic phosphors or other luminescent materials for a wide range of applications. As processes such as energy transfer strongly depend on the distance between donor and acceptor, and charge transfer may likewise exhibit a distance dependence, it would be highly informative to perform these measurements on single crystals with varying dopant concentrations as well.

## Materials and methods

The SrAl_2_O_4_:Eu (300 ± 100 ppm Eu) and SrAl_2_O_4_:Eu, Dy (500 ± 100 ppm Eu, 50 ± 10 ppm Dy) single crystals were obtained by a procedure developed by BREVALOR and were grown by pulling from the melt using a Czochralski-type process.

In all experiments, the temperature of the sample was controlled using a Linkam FTIRSP600 stage equipped with an LNP96 unit to cool below room temperature using liquid nitrogen. The stage was used to measure in both transmission (for transient absorption measurements) and reflection geometry (for luminescence lifetime, thermoluminescence and thermal quenching measurements).

Afterglow curves were recorded after 5 min of illumination with 1000 lux of white light from a Xe-arc lamp using a calibrated ILT 1700 detector equipped with a photopic YPM filter.

Luminescence lifetimes were measured using a 1024-channel intensified CCD (Andor Technology) attached to a 0.5 m Ebert monochromator. An Ekspla NT340 wavelength-tunable OPO laser was used as a pulsed excitation source. The power per pulse was determined using an ES220C pyroelectric sensor coupled to a PM400 power meter from Thorlabs. The thermal quenching and thermoluminescence spectra were recorded using a fiber-coupled spectrometer (AvaSpec-HERO, 195–980 nm, Avantes).

Transient absorption spectroscopy was performed via a home-built setup that uses an Yb:KGW based laser (Light Conversion Carbide 40 W) as central element. It outputs pulses at variable repetition rates, where 1 kHz is used for the experiments in this paper. The pulses have a duration of 192 fs, as measured via autocorrelation, and are centered at 1030 nm. The green (515 nm) and the UV (343 nm) pumps are obtained via second and third harmonic generation, respectively. For second harmonic generation, a BBO crystal matched to the pulse duration of 220 fs is used (EKSMA Optics), whereas the 3rd harmonic requires additionally a third harmonic generation BBO crystal (EKSMA Optics) combined with a calcite timing plate (EKSMA Optics) and a wave plate (EKSMA Optics). The blue pump (430 nm) is obtained via an optical parametric amplifier (NEO-HP, Light Conversion). Pump pulses are mechanically chopped (Newport) to obtain a differential transmission signal. The probe pulse is timed via a mechanical delay stage (Newport MKS, DL325) and spectrally broadened via supercontinuum generation in a 10 mm thick YAG crystal. The probe spectrum is detected with a Si (visible wavelength range) or InGaAs (NIR range) detector coupled to a home-built grating spectrometer. Read-out electronics and software have been built in-house. The noise floor for the Δ*A* in this setup amounts to 20–30 µOD.

The power of continuous illumination sources used during transient absorption and luminescence lifetime measurements was measured using a S401C thermal power sensor coupled to a PM400 power meter from Thorlabs.

## Supplementary information

Supplementary information

## Data Availability

The data that support the findings of this study are available from the corresponding author upon request.
