# Supplementary material for: A direct observation of ultrafast energy transfer, charge trapping and thermal quenching in inorganic phosphors
Source: Light Sci Appl. 2026 Oct 27;15:391. doi: 10.1038/s41377-026-02433-z (PMC13714306; doi:10.1038/s41377-026-02433-z)
Supplement: Supplementary file 1 [file 41377_2026_2433_MOESM1_ESM.pdf]

Supplementary Information for "A direct observation of ultrafast energy transfer, charge trapping and thermal quenching in inorganic phosphors"

David Van der Heggen, Korneel Molkens, Jonas J. Joos,
Daniel Rytz, Lucas C.V. Rodrigues, Pieter Geiregat, Philippe F. Smet

Fitting of transient absorption measurements with a UV (343 nm) pump on $SrAl_2O_4$:Eu,Dy at different temperatures

The transient absorption time traces $\Delta A(700\text{ nm}, t)$ obtained using a UV (343 nm) pump pulse on $SrAl_2O_4$:Eu,Dy at different temperatures (Fig. 2f) exhibit an exponential rise (due to energy transfer from Eu^{2+}_{SrI} to Eu^{2+}_{SrII} centers) and an exponential decay (due to charge transfer from Eu^{2+} to a trapping defect) in absorbance. The data was fit using the following prescription:

$$I(t) = A_1 \left(e^{-t/\tau_1} - \alpha\, e^{-t/\tau_2}\right) \tag{1}$$

where A_1 and α are scaling factors, τ_1 corresponds to the decay time and τ_2 is the rise time. The results of the fitting procedure are shown in Fig. 2f and summarized in Table S1.

T (K)	τ_1 (ns)	τ_2 (ps)
293	-	510
325	-	63
350	17	21
375	8.1	9.6
400	3.1	4.3
425	1.0	2.7
475	0.18	1.9
525	0.068	1.7

Table S1: The transient absorption decay time (τ_1) and rise time (τ_2) extracted from fitting the transient absorption time traces $\Delta A(700\text{ nm}, t)$ obtained using a UV (343 nm) pump pulse on $SrAl_2O_4$:Eu,Dy at different temperatures.

Fitting of transient absorption measurements with a UV (343 nm) pump on $SrAl_2O_4$:Eu at different temperatures

The transient absorption time traces $\Delta A(700\text{ nm}, t)$ obtained using a UV (343 nm) pump pulse on $SrAl_2O_4$:Eu at different temperatures (Fig. 2b) exhibit an exponential rise (due to energy transfer from Eu^{2+}_{SrI} to Eu^{2+}_{SrII} centers) and two distinct phases of exponential decay (due to quenching of the Eu^{2+}_{SrI} and Eu^{2+}_{SrII} excited states) in absorbance. The data was fit using the following prescription:

$$I(t) = A_1\, e^{-t/\tau_1} + A_2 \left(e^{-t/\tau_2} - \alpha\, e^{-t/\tau_3}\right) \tag{2}$$

where A_1, A_2 and α are scaling factors. τ_1 corresponds to the decay time associated with the quenching of the Eu^{2+}_{SrI} excited state, τ_2 corresponds to the decay time associated with the quenching of the Eu^{2+}_{SrII} excited state and τ_3 is the rise time associated with the energy transfer. If a component is not present at a certain temperature, the corresponding prefactor was set to 0 during the fitting procedure. The results of the fitting procedure are shown in Fig. 2b and summarized in Table S2.

Photoluminescence lifetime measurements at different temperatures

The photoluminescence lifetimes (Fig. 3c) of the blue Eu^{2+}_{SrI} emission were obtained by fitting the photoluminescence decay curves (Fig. 3b) using a bi-exponential decay:

$$I(t) = A_1\, e^{-t/\tau_1} + A_2\, e^{-t/\tau_2} + C \tag{3}$$

where A_i is a prefactor that corresponds to the initial intensity, τ_i is the photoluminescence lifetime and C is a constant that accounts for the persistent luminescence of the material which has a constant intensity at these short timescales. The results of the fits are shown in Fig. 3b and summarized in Table S3. The contribution of each component to the overall luminescence intensity was calculated using:

$$f_i = \frac{A_i \tau_i}{\sum_j A_j \tau_j} \tag{4}$$

T (K)	τ_1 (ps)	τ_2 (ns)	τ_3 (ps)
293	-	-	714
325	-	-	154
350	-	-	43
375	-	-	19
400	-	-	11
425	1.8	193	8.2
475	2.4	97	5.4
525	1.1	34	6.2
575	0.83	18	6.1
625	0.77	10	4.6

Table S2: The transient absorption decay times (τ_1 and τ_2) and rise time (τ_3) extracted from fitting the transient absorption time traces $\Delta A(700 \text{ nm}, t)$ obtained using a UV (343 nm) pump pulse on $SrAl_2O_4$:Eu at different temperatures.

T (K)	τ_1 (ns)	f_1 (%)	τ_2 (ns)	f_2 (%)
75	753	87	234	13
125	811	72	292	28
175	426	76	991	24
225	212	79	1007	21

Table S3: Photoluminescence lifetimes (τ_i) of the blue Eu^{2+}_{SrI} emission under 410 nm excitation. The corresponding fractions (f_i) are calculated using Equation 4.

T (K)	τ_1 (ns)	f_1 (%)	τ_2 (ns)	f_2 (%) or α	Prescription
75	1094	100	847	0.65	Eq. 5
125	1177	100	688	0.37	Eq. 5
175	1202	100	510	0.24	Eq. 5
225	1174	100	-	-	Eq. 5
275	526	84	1403	16	Eq. 3
293	213	76	783	24	Eq. 3
348	20	100	-	-	Eq. 3

Table S4: Photoluminescence lifetimes of the green Eu^{2+}_{SrII} emission under 410 nm excitation. Please refer to the text for the definition of τ_2 at various temperatures. The corresponding fractions (f_i) are calculated using Equation 4.

where f_i is the fraction of the emission intensity belonging to the component with decay time τ_i.
The photoluminescence lifetimes of the green Eu^{2+}_{SrII} emission (Fig. 3c) were obtained by fitting the photoluminescence decay curves (Fig. 3a) with two different prescriptions. For temperatures up to 225 K the energy transfer from the Eu^{2+}_{SrI} to the Eu^{2+}_{SrII} centers results in an intensity increase in the initial part of the decay. Hence, the decay curves were fitted using the following prescription:

$$I(t) = A_1 \left(e^{-t/\tau_1} - \alpha\, e^{-t/\tau_2}\right) + C \tag{5}$$

where A_1 and α are scaling factors, τ_1 is the photoluminescence lifetime of the green Eu^{2+}_{SrII} emission, τ_2 is the luminescence rise time and C is a constant that accounts for the persistent luminescence of the material. The value of τ_2 was constrained between 90 - 110 % of the Eu^{2+}_{SrI} photoluminescence lifetime listed in Table S3. At higher temperatures the luminescence decay curves were fitted using Equation 3. The results of the fits are shown in Fig. 3a and summarized in Table S4.

Intensity dependent photoluminescence and optically stimulated luminescence lifetime measurements

The Eu^{2+}_{SrII} lifetimes in the $SrAl_2O_4$:Eu,Dy single crystal were recorded under pulsed excitation (410 nm, 10 Hz) for different excitation pulse energies. The obtained decay curves were fitted using Equation 6

where one exponential (lifetime τ_{bck}) was used to fit the background which is due to the afterglow of the single crystal. The fitting results for the two fast components (τ_1 and τ_2) are summarized in Table S5.

$$I(t) = A_1\ e^{-t/\tau_1} + A_2\ e^{-t/\tau_2} + A_{\text{bck}}\ e^{-t/\tau_{\text{bck}}} \tag{6}$$

E(mJ cm^{-2})	τ_1 (ns)	f_1 (%)	τ_2 (ns)	f_2 (%)
124	114	80	983	20
23.8	170	72	961	28
4.3	179	76	891	24
0.6	196	87	795	13
0.12	100	86	350	13
0.023	10	99	128	1

Table S5: Lifetimes (τ_i) of the green Eu^{2+}_{SrII} emission under pulsed excitation with 410 nm light (10 Hz). The corresponding fractions (f_i) are calculated using Eq. 4, excluding the component accounting for the background.

The Eu^{2+}_{SrII} lifetimes in the $SrAl_2O_4$:Eu,Dy single crystal were also recorded under pulsed stimulation (520 nm, 10 Hz) in combination with continuous 405 nm excitation to keep the traps filled. The obtained decay curves were fitted using Equation 6 where one exponential again accounts for the background. The fitting results are summarized in Table S6.

E(mJ cm^{-2})	τ_1 (ns)	f_1 (%)	τ_2 (ns)	f_2 (%)
High 405 nm excitation intensity: 833 mW cm^{-2}				
330	110	36	416	64
71.8	127	27	487	73
15.3	137	20	553	80
1.4	164	14	648	86
0.5	144	12	694	88
Medium 405 nm excitation intensity: 78 mW cm^{-2}				
272	111	44	387	56
84.5	130	36	443	64
13.4	137	26	503	74
2.0	171	19	573	81
Low 405 nm excitation intensity: 5 mW cm^{-2}				
360	96	69	293	31
66.0	109	60	333	40
18.8	119	52	361	48
1.8	145	42	424	58

Table S6: Lifetimes (τ_i) of the green Eu^{2+}_{SrII} emission under continuous excitation with 405 nm light of different intensities and pulsed stimulation with different intensities of 520 nm light (10 Hz). The corresponding fractions (f_i) are calculated using Equation 4, excluding the component accounting for the background.

Simulated excited state absorption spectrum of Eu^{2+}

Excited-state absorption spectra originating from the lowest level of the $4f^6 5d^1$ configuration, which has a radiative lifetime of approximately 1 µs, were simulated for the Eu_{SrI} and Eu_{SrII} defects. The final states of the electric-dipole-allowed transitions correspond to the excited multiplets of the $4f^7$ configuration, in particular the ^6P_J ($J = 7/2, 5/2, 3/2$), ^6I_J ($J = 17/2, \ldots, 7/2$), and ^6D_J ($J = 9/2, \ldots, 1/2$), located at approximately 28 000 cm^{-1}, 31 000 cm^{-1}, and 34 000 cm^{-1} above the $4f^7(^8S_{7/2})$ ground state, respectively.

For these simulations, *ab initio* multiplet energies of Eu^{2+} in CaF_2 reported in Ref.[1] were employed, under the assumption that these energies are only weakly dependent on the host lattice. Experimental values from Ref.[2] were used for the lowest level of the $4f^6 5d^1$ configuration. To construct the final spectra, a Huang–Rhys–Pekar envelope was applied to each electronic transition, using electron–phonon coupling parameters experimentally extracted from $4f^7(^8S_{7/2}) \leftrightarrow 4f^6 5d^1$ photoluminescence spectra.[2,3] Since no information on individual oscillator strengths is available, all transitions were assigned equal weight, which may affect the overall spectral profile. Given the approximations involved, the simulated spectra should be regarded as strictly qualitative.

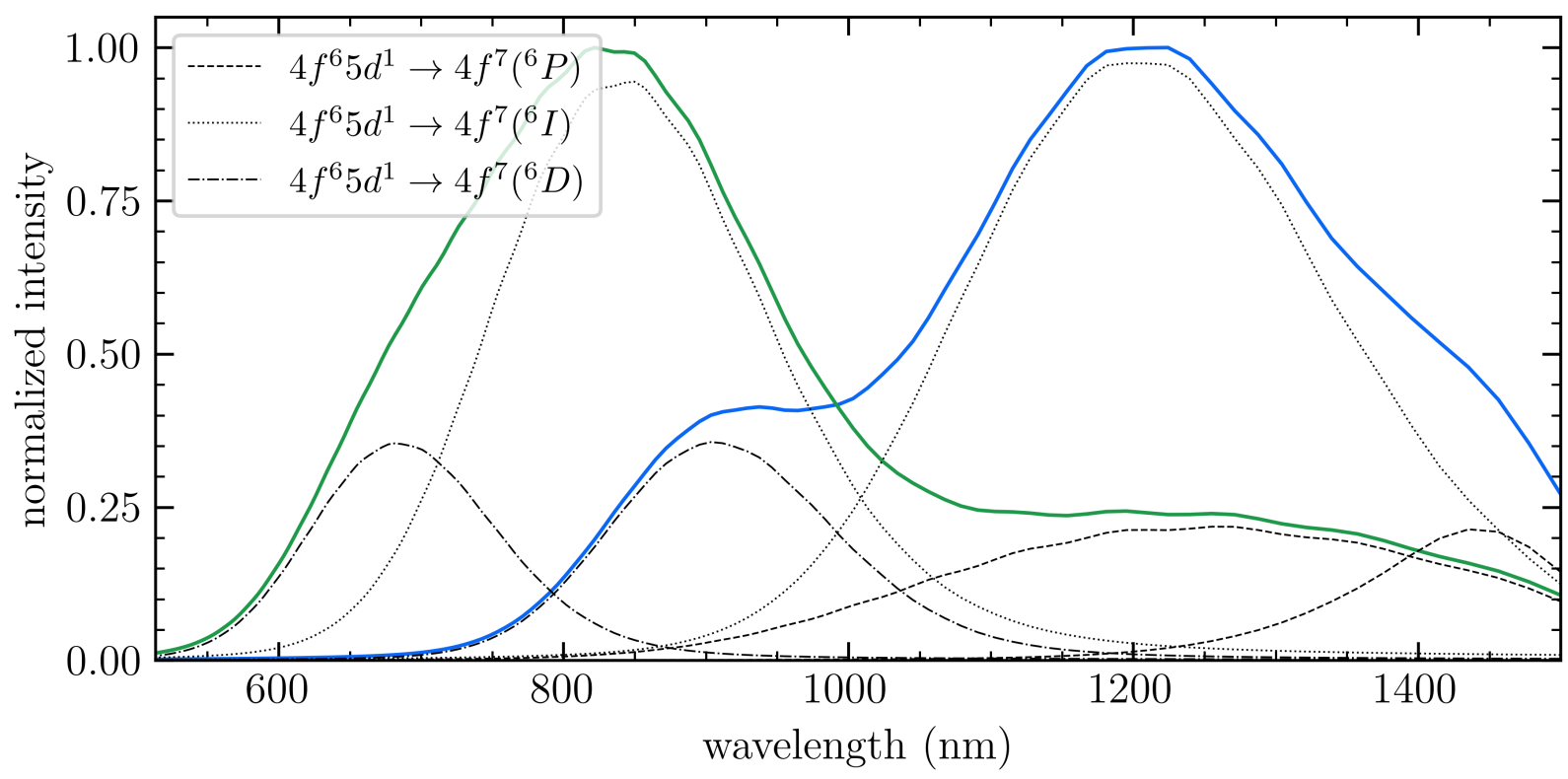

Fig. S1: Simulated excited state absorption spectrum for Eu_{SrI} centers (blue curve) and Eu_{SrII} centers (green curve) in $SrAl_2O_4$.

Thermoluminescence measurements

To investigate the thermal activation of charge trapping in the $SrAl_2O_4$:Eu,Dy single crystal it was exposed to 410 nm light at various temperatures. Immediately following this excitation the samples were heated at a linear rate (30 °C min^{-1}) and the resulting thermoluminescence was recorded. The results are shown in Fig. S2 and all glow curves were corrected for thermal quenching. The thermoluminescence glow curves consist of two components centered around 260 K and 325 K. The total thermoluminescence intensity increases with charging temperature. As the charging temperature increases an increasingly large fraction of the intensity is emitted as afterglow immediately after the charging has stopped. The integrated thermoluminescence intensity as a function of excitation temperature can be found in Fig. 4c.

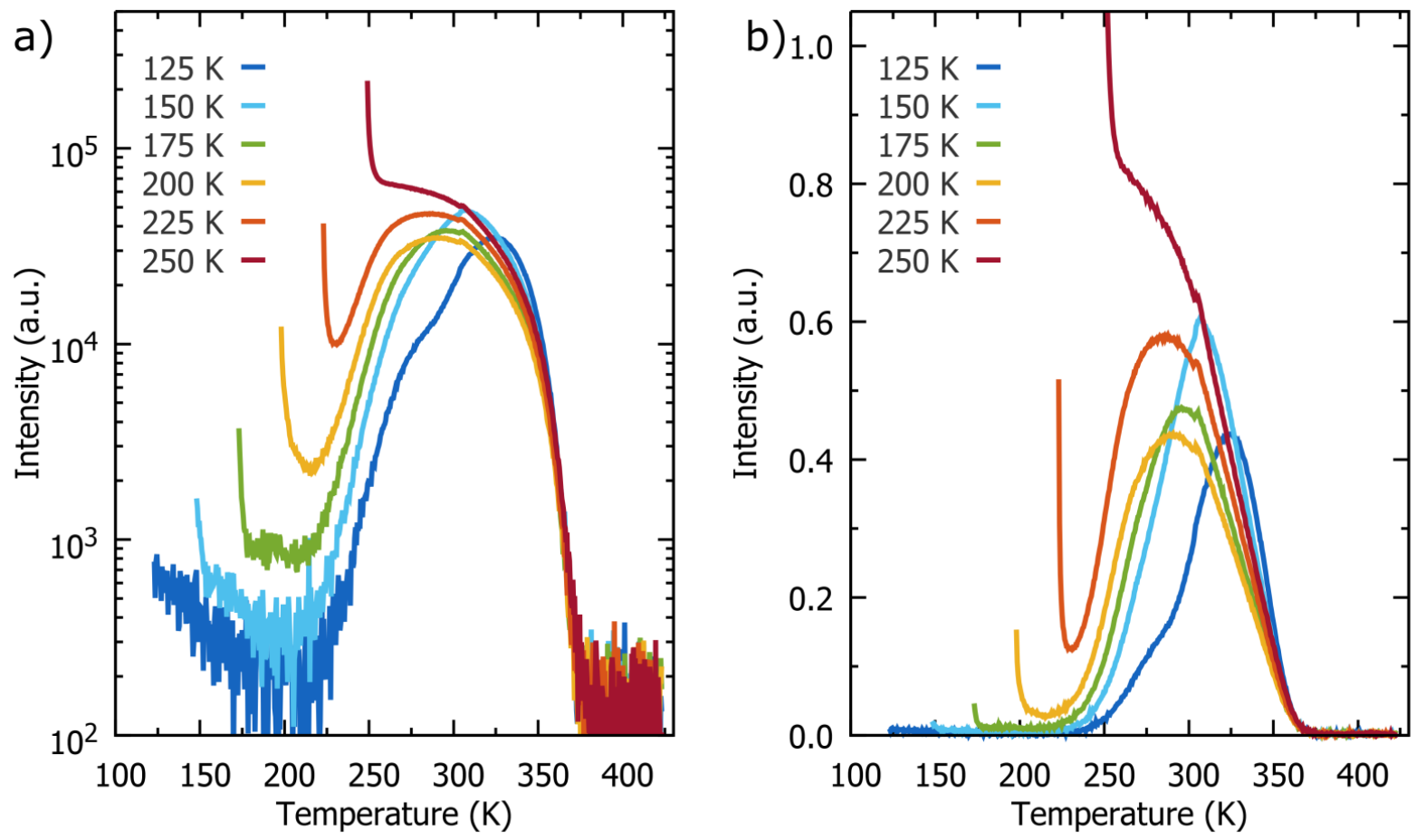

Fig. S2: Thermoluminescence glow curves recorded on the $SrAl_2O_4$:Eu,Dy single crystal after excitation with 410 nm light at different temperatures and shown on a) a logarithmic scale and b) a linear scale.

Dy^{2+} absorption spectrum

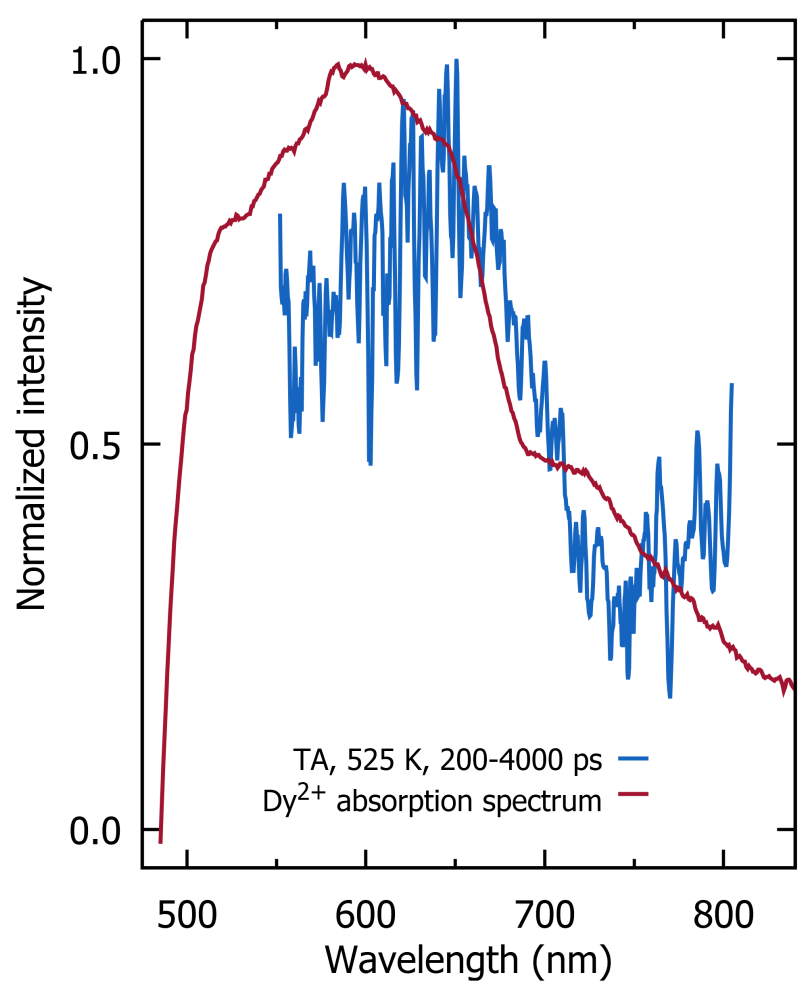

Fig. S3: Comparison of the absorption spectrum[4] of Dy^{2+} in $SrAl_2O_4$ recorded at 75 K and the absorption spectrum obtained 200 ps after excitation with a UV (343 nm) pump at 525 K.

Transient absorption spectroscopy with a green pump

To investigate the optically stimulated depopulation of previously trapped charges and the subsequent charge transfer from Dy^{2+} to Eu^{3+}, transient absorption measurements were carried out using a green pump pulse (515 nm). To ensure a constant population of trapped charges during the experiment, the single crystal was continuously illuminated with a blue LED (420 nm), operated at varying drive currents. In the absence of blue illumination (0 mA), trapped charges are not present; consequently, the green pump pulse does not induce any measurable change in absorption. The sharp feature at $t = 0$ ps is most likely due to an optical Kerr effect in the crystal, marking the temporal overlap of the pump and probe pulses.

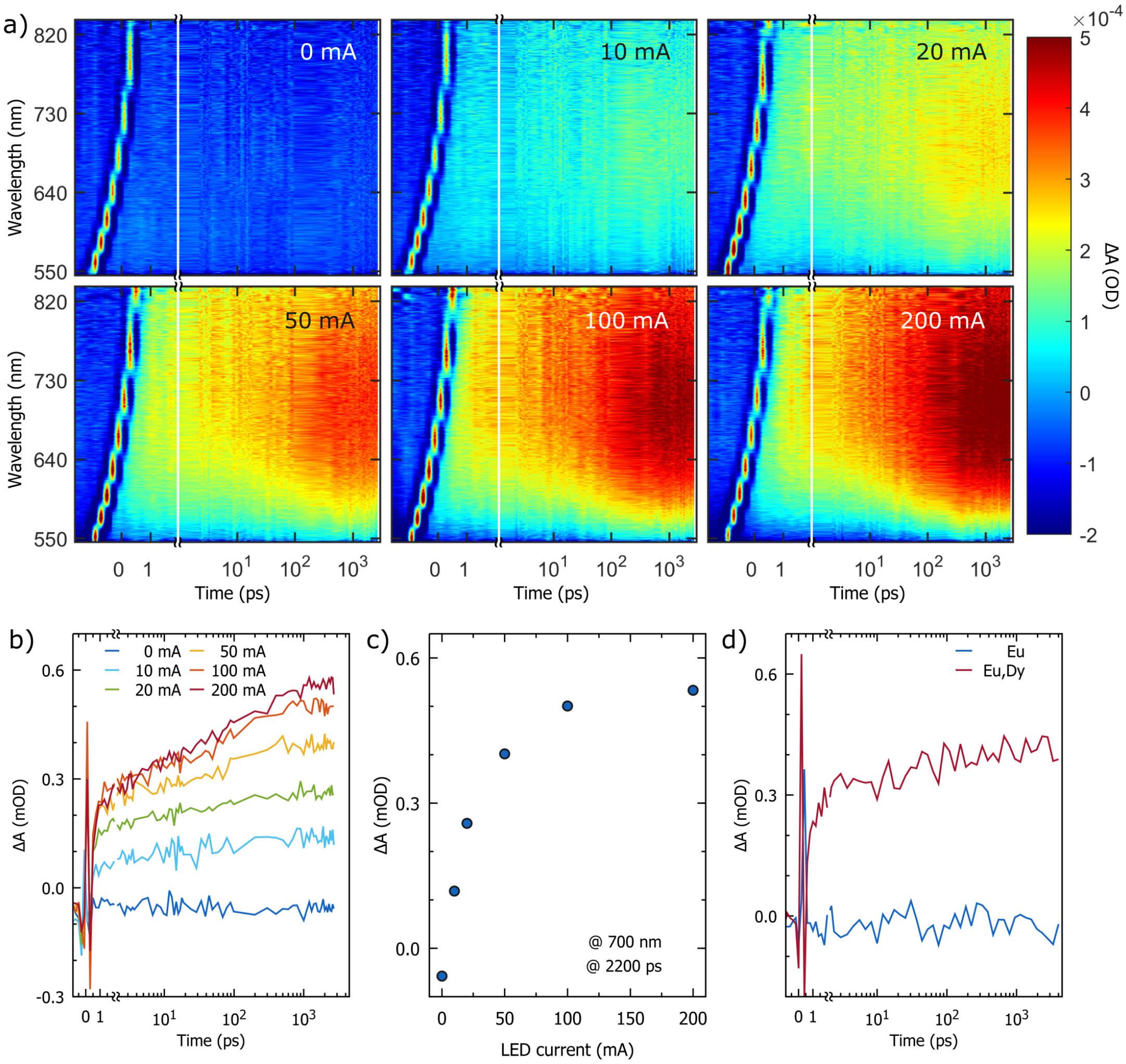

Fig. S4: Transient absorption of the $SrAl_2O_4$:Eu and $SrAl_2O_4$:Eu,Dy single crystals recorded using a green pump (515 nm, 946 µJ cm^{-2}) under continuous illumination with blue 420 nm light at room temperature. (a) Color maps showing the resulting $\Delta A(\lambda,t)$ recorded on $SrAl_2O_4$:Eu,Dy at 300 K under different intensities of continuous blue illumination. (b) Time traces $\Delta A(700\text{ nm}, t)$ recorded under different intensities of continuous blue illumination. (c) $\Delta A(700\text{ nm}, 2\,200\text{ ps})$ as a function of the blue light intensity. (d) Time traces $\Delta A(700\text{ nm}, t)$ recorded on the $SrAl_2O_4$:Eu and $SrAl_2O_4$:Eu,Dy single crystals under illumination with blue light (410 nm, 200 mA).

Comparison of transient absorption spectroscopy with a UV (343 nm) and green (515 nm) pump

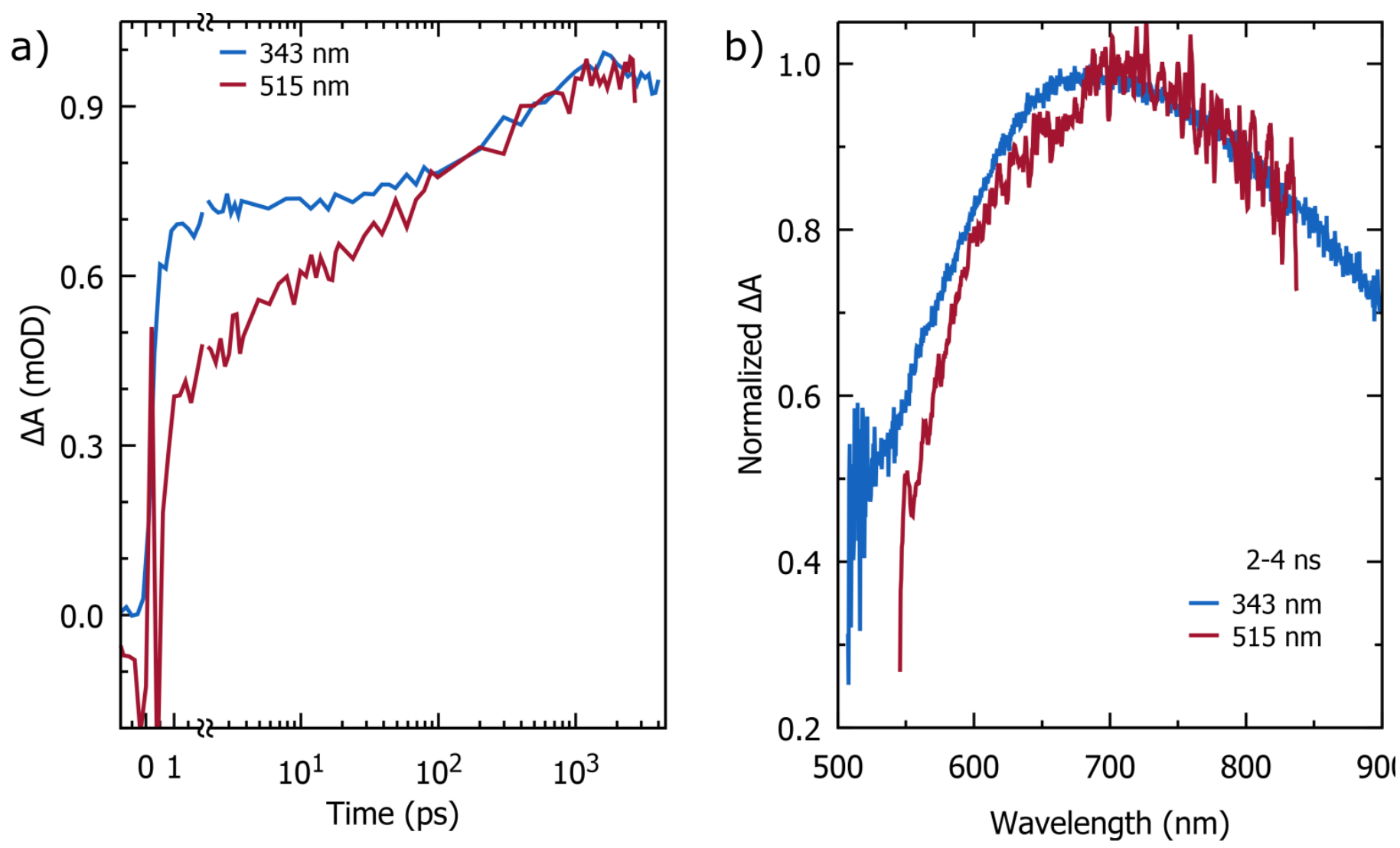

Fig. S5: Comparison of the transient absorption recorded at room temperature on the $SrAl_2O_4$:Eu,Dy single crystals using a UV and a green pulse. a) Time traces showing $\Delta A(700\text{ nm}, t)$. Both curves have been horizontally aligned so that the pump pulse reaches the sample at $t = 0$ ps. b) Average absorbance spectra recorded 2-4 ns after the pump pulse.

Photoluminescence lifetime measurements under high excitation intensity at different temperatures

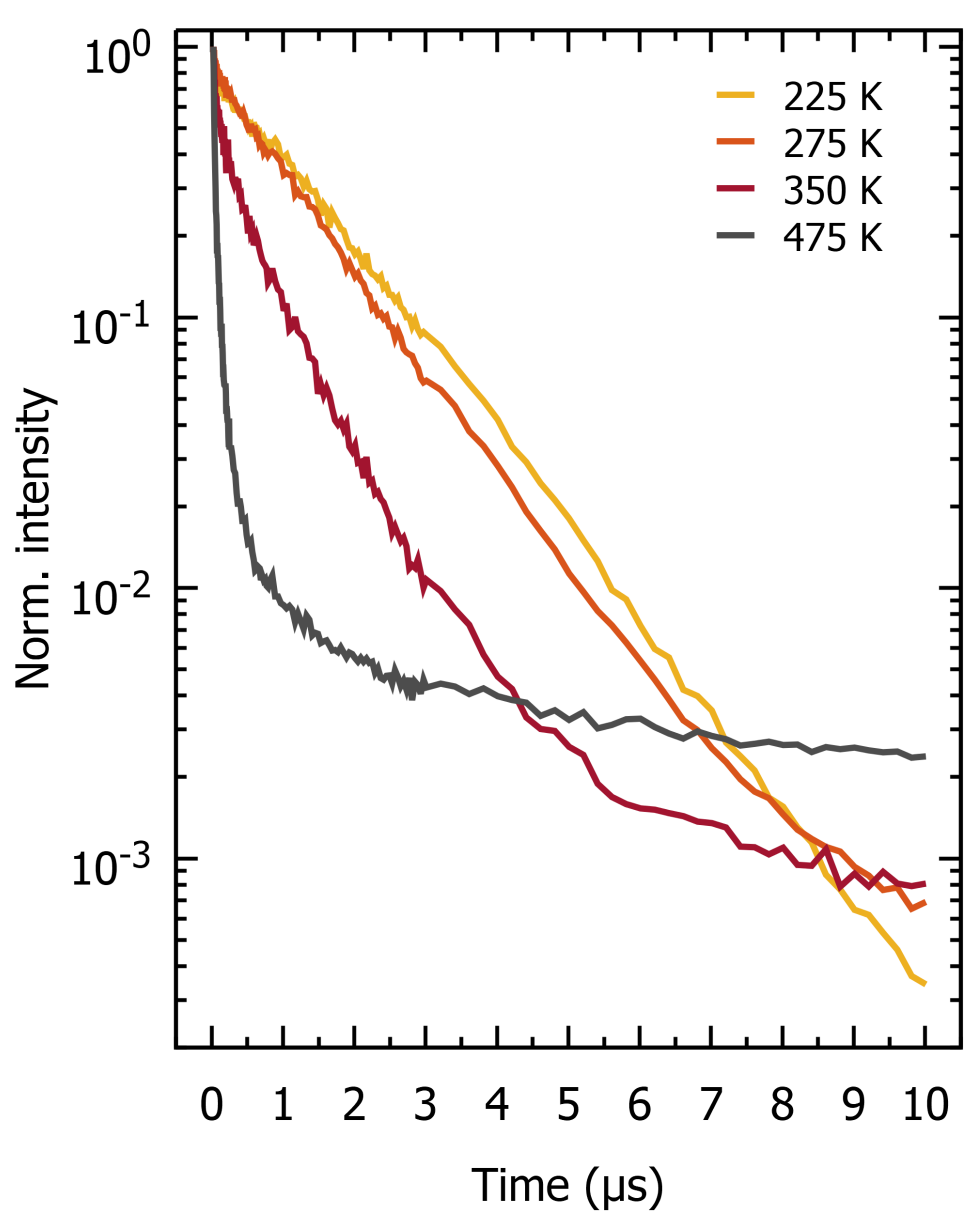

Fig. S6: Photoluminescence decay curves of the green emission of the $SrAl_2O_4$:Eu,Dy single crystal at different temperatures after excitation with a high intensity (25.8 mJ cm^{-2}, 0.5 Hz) 410 nm excitation pulse.

Calculating the spectral overlap for energy transfer

Calculating the spectral overlap integral in the entire temperature range (75 - 550 K) is not directly possible from the experimental emission and excitation spectra; the donor emission gets rapidly quenched when the temperature increases and obtaining reliable acceptor excitation spectra is impossible except at low temperatures due to afterglow effects. Estimating the overlap integral can hence only be done by extrapolating the spectra. For this, Huang-Rhys-Pekar envelopes were fitted at the experimental spectra in the low temperature range;[5] for the donor emission, this could be reliably done by one envelope, for the acceptor excitation spectrum, consisting of a very high number of individual electronic transitions, a satisfactory result for the low-energy tail could be achieved by combining three envelopes. When the fit was optimized, the temperature-induced broadening, and hence the temperature dependence of the spectra overlap, was calculated by extrapolating the temperature parameter in the Huang-Rhys-Pekar ansatz up to 550 K.

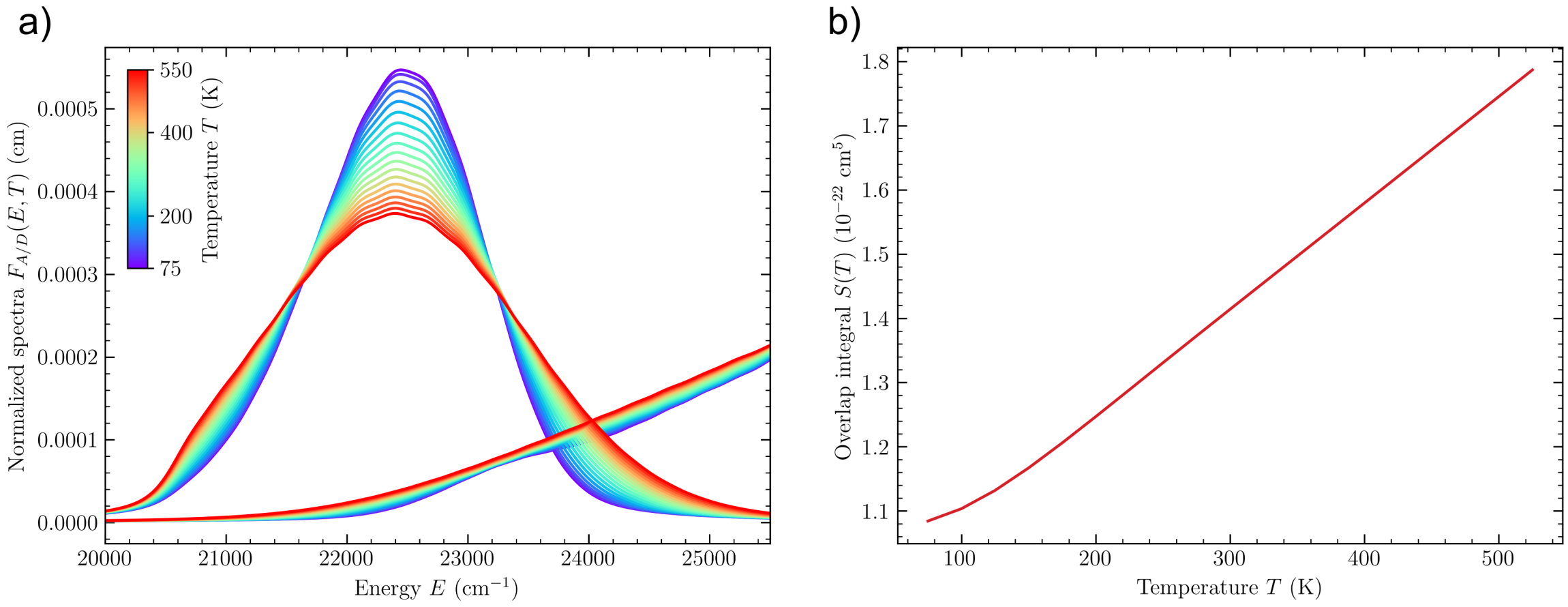

Fig. S7: a) The overlap between the extrapolated Eu^{2+}_{SrI} emission spectrum and the low energy tail of the Eu^{2+}_{SrII} excitation spectrum at various temperatures. b) The calculated overlap integral as a function of temperature.

Comparison of afterglow intensities of $SrAl_2O_4$:Eu and $SrAl_2O_4$:Eu,Dy crystals

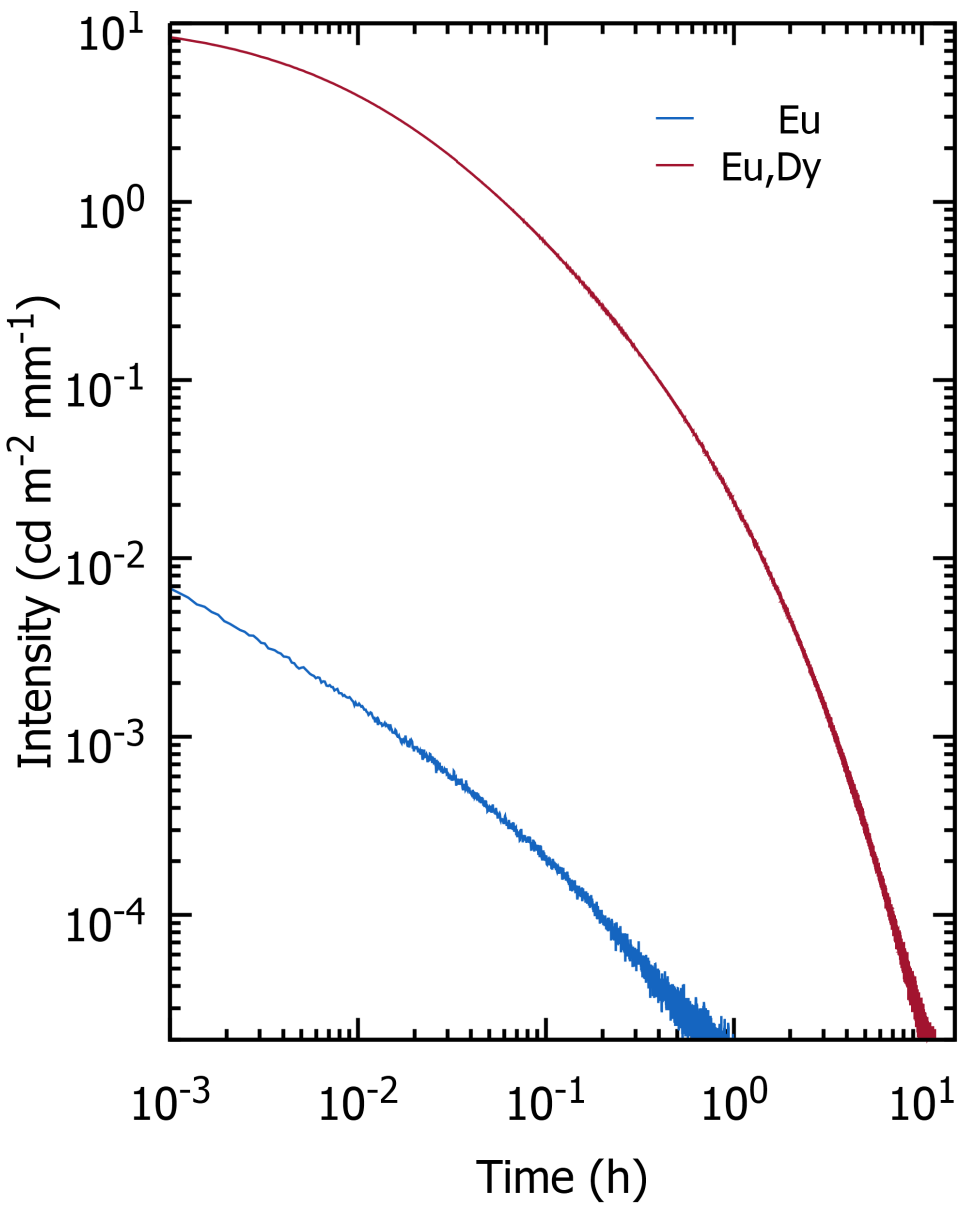

Fig. S8: Afterglow curves (corrected for single crystal thickness) of the $SrAl_2O_4$:Eu and $SrAl_2O_4$:Eu,Dy single crystals recorded at room temperature after 5 minutes of illumination with 1000 lux of white light from a Xe-arc lamp.

References

[1] Joos, J. J., et al. Insights into the Complexity of the Excited States of Eu-Doped Luminescent Materials. *Inorganic Chemistry Frontiers* **7**, 871–888 (2020).

[2] Botterman, J., Joos, J. J. & Smet, P. F. Trapping and detrapping in $SrAl_2O_4$:Eu,Dy persistent phosphors: Influence of excitation wavelength and temperature. *Physical Review B - Condensed Matter and Materials Physics* **90**, 085147 (2014).

[3] Nazarov, M. *et al.* Phonon-assisted optical bands of nanosized powdery $SrAl_2O_4$:Eu^{2+} crystals: Evidence of a multimode Pekarian. *Physics Letters A* **377**, 3170–3178 (2013).

[4] Van der Heggen, D., Joos, J. J. & Smet, P. F. Optical Evidence of Charge Trapping at Lanthanide Codopants in $SrAl_2O_4$:Eu,Ln (Ln = Nd, Sm, Dy) Persistent Phosphors. *Advanced Optical Materials* **13**, 2402153 (2025).

[5] Huang, K. & Rhys, A. Theory of Light Absorption and Non-Radiative Transitions in F-Centres. *Proceedings of the Royal Society of London A: Mathematical, Physical and Engineering Sciences* **204**, 406–423 (1950).
